# Computed tomography of the equine caudal spine and pelvis: Technique, image quality and anatomical variation in 56 clinical cases (2018–2023)

**DOI:** 10.1111/evj.14422

**Published:** 2024-10-10

**Authors:** Nadine Kristina Elise Ogden, Katja Winderickx, Alison Bennell, John David Stack

**Affiliations:** ^1^ B&W Equine Hospital Berkeley UK; ^2^ Lingehoeve Diergeneeskunde Lienden Netherlands; ^3^ Leahurst Equine Hospital, University of Liverpool Wirral UK

**Keywords:** CT, horse, lumbosacral, pelvis, sacroiliac

## Abstract

**Background:**

Cross‐sectional imaging improves the diagnostic accuracy of complex anatomical regions. Computed tomography (CT) of the pelvis and caudal spine in a large group of live horses and ponies has not been previously reported.

**Objective:**

To describe the procedure for acquiring CT images of horses' caudal spine/pelvis under general anaesthesia (GA) and to detail the image quality, artefacts and anatomical variations in this region.

**Study design:**

Retrospective case series.

**Methods:**

Horses with CT of the caudal spine/pelvis were included. Horses under 6 months and CT examination performed *post‐mortem* were excluded. Protocols, image quality, region of interest, anatomical features and morbidities were analysed.

**Results:**

Fifty‐six horses (8 months to 20 years, 85–680 kg) met the inclusion criteria. GA ranged from 10 to 60 min (mean: 30, median: 32). There were no adverse events recorded in any of the horses associated with the procedure. Images of all horses were considered of diagnostic quality. Anatomical variations were common and included the location of diverging (widest) interspinous space, the presence of spina bifida in the lumbar and sacral spine, the shape of the last lumbar vertebra and the location of intertransverse joints in terms of where they were present and the degree of fusion/modelling.

**Main limitations:**

Not all horses underwent CT examination of the same regions, the upper size limit of horses is unknown and will vary depending on bore size and table infrastructure. Image noise, particularly in large horses and beam hardening artefacts from hardware and pelvis degraded image quality. Images were of insufficient quality in large horses for soft tissue interpretation.

**Conclusion:**

CT of the caudal spine and pelvis in live horses with wide‐bore CT machines and modified patient infrastructure was safe and produced diagnostic images.

## Abstract (de)

**Hintergrund:**

Die Querschnittsbildgebung verbessert die diagnostische Genauigkeit von komplexen anatomischen Regionen. Die Computertomographie (CT) des Beckens und der kaudalen Wirbelsäule bei einer groβen Gruppe lebender Pferde und Ponys wurde bisher noch nicht berichtet.

**Ziel der Studie:**

Beschreibung des Verfahrens zur Aufnahme von CT‐Bildern der kaudalen Wirbelsäule/des Beckens von Pferden unter Vollnarkose und detaillierte Beschreibung der Bildqualität, Artefakte und anatomischen Variationen in diesen Regionen.

**Studiendesign:**

Retrospektive Fallserie.

**Methodik:**

Pferde mit CT der kaudalen Wirbelsäule/des Beckens wurden in die Studie aufgenommen. Pferde, die jünger als 6 Monate waren und bei denen die CT‐Untersuchung postmortal durchgeführt wurde, wurden ausgeschlossen. Analysiert wurden Protokolle, Bildqualität, Interessengebiet, anatomische Merkmale und Morbiditäten.

**Ergebnisse:**

Sechsundfünfzig Pferde (8 Monate bis 20 Jahre, 85–680 kg) erfüllten die Einschlusskriterien. Die Dauer der Vollnarkose (GA) lag zwischen 10 und 60 Minuten (Mittelwert 30, Median 32). Bei keinem der Pferde wurden unerwünschte Ereignisse im Zusammenhang mit dem Verfahren festgestellt. Die Bilder aller Pferde waren von diagnostischer Qualität. Anatomische Abweichungen waren häufig und betrafen u.a.: die Lage des divergierenden (breitesten) Zwischenwirbelraums, das Vorhandensein einer Spina bifida in der Lenden‐ und Sakralwirbelsäule, die Form des letzten Lendenwirbels und die Lage der Intertransversalgelenke in Bezug auf deren Vorhandensein und den Grad der Fusion/Modellierung.

**Haupteinschränkungen:**

Nicht bei allen Pferden wurden die gleichen Regionen im CT untersucht, die Obergrenze für die Gröβe der Pferde ist unbekannt und hängt von dem Gantrydurchmessers und der Tischkonstruktion ab. Bildrauschen, insbesondere bei groβen Pferden, und Strahlenhärtungsartefakte von Hardware und Becken beeinträchtigten die Bildqualität für die Interpretation von Weichteilen.

**Schlussfolgerung:**

Die Computertomographie der kaudalen Wirbelsäule und des Beckens bei lebenden Pferden mit Wide‐Bore‐CT‐Geräten und modifizierter Patienteninfrastuktur war sicher und lieferte diagnostische Bilder.

## 1 INTRODUCTION

Computed tomography (CT) is becoming more readily available for horses. It has quickly become the accepted gold standard diagnostic imaging modality for complex anatomical regions such as the head and cervical spine.^1^, ^2^, ^3^ Technological advances facilitating this rapid adoption of CT imaging included the advent of CT machines with wide bores (80–90 cm), sliding gantries and custom‐built patient tables and platforms that can accommodate horses and facilitate positioning.

The anatomy of the caudal spine and pelvis in horses is complex and commonly affected by anatomical and congenital variabilities and degenerative changes.^4^, ^5^, ^6^ The relationship between these structural differences, biomechanics and aetiopathogenesis of disease is poorly understood and highlights the requirement for clinical studies. Traditional diagnostic imaging modalities of the caudal spine and pelvis in horses offer only limited diagnostic information. Skeletal scintigraphy of the pelvic region of lame or poorly performing horses produces both false‐positive and false‐negative results.^7^, ^8^ Radiography of the sacroiliac joints has been described in horses under general anaesthesia (GA) but has not been adopted clinically due to the limited amount of diagnostic data obtained^9^ and standing radiography of the coxofemoral joint is feasible but provides limited information on the lesion type and configuration.^10^ Both percutaneous and transrectal ultrasonography scanning can be used in horses to image substantial parts of the surface of the lumbosacroiliac and pelvic regions with the identification of fracture and osteoarthritis; however, ultrasonography has limited capability to image bone and not all aspects can be imaged.^6^, ^9^, ^11^, ^12^, ^13^, ^14^, ^15^ Due to the limitations of radiography and ultrasonography in assessing the caudal spine and pelvis, these imaging modalities have been superseded by CT and MRI in humans and small animals.^16^, ^17^, ^18^ The superiority of CT over traditional imaging modalities for this region has been documented in foals with caudal spine and pelvic disease.^19^ Co of dissected and eviscerated adult caudal spine and pelvic specimens has been reported in the literature.^3^, ^4^, ^6^, ^20^, ^21^ However, caudal spine and pelvic CT has not been reported in live full‐size adult horses due to the practical limitations of patient positioning including gantry diameter and weight‐restricted CT tables. CT protocols have not been published for live horses undergoing caudal spine and pelvis CT scanning.

This case series describes the techniques and protocols for performing CT of the caudal spine and pelvis of live horses and ponies over 6 months of age and reports the anatomical and congenital findings in these horses. We anticipated that CT of the caudal spine and pelvis in horses and ponies, performed under GA, would have a low morbidity and mortality and, that diagnostic‐quality images would be able to be obtained and that a range of anatomical variations would be detected/noted within this region.

## 2 MATERIALS AND METHODS

### 2.1 Study subjects

In this retrospective case series, horses with CT examination of the caudal spine and/or pelvis at Philip Leverhulme Equine Hospital (University of Liverpool) (Hospital 1) and Lingehoeve Diergeneeskunde (Hospital 2), between November 2018 and April 2023, were included. For this study, ‘caudal spine’ is defined as the caudal thoracic vertebra (T16–18), the lumbar vertebra and sacrum. Horses and ponies were included if they had part or all the caudal spine and part or all of the pelvis included in the field of view (FOV). Horses aged under 6 months and CT examinations that had been performed *post‐mortem* were excluded.

Data retrieved from the medical records included horse signalment, history, presenting signs, clinical and imaging diagnosis, anaesthesia time and adverse events during the CT procedure and recovery. In horses where medical records are incomplete, the missing data was reported. Pathological CT imaging findings for these horses and ponies have been reported in a separate study.^22^

### 2.2 CT examination

#### 2.2.1 Anaesthesia

At Hospital 1, horses all underwent premedication with acepromazine maleate (AceSedate, Zoetis UK Limited) (0.03–0.05 mg/kg bwt im or iv), after performing a thorough clinical examination. Horses then received additional premedication with romifidine (Sedivet, Boehringer Ingelheim Animal Health UK Ltd) (50–100 mcg/kg iv) and morphine sulphate (Morphine Sulfate; Wockhardt UK Ltd) or methadone (Comfortan; Dechra Veterinary Products) (0.1–0.2 mg/kg bwt iv), with the opioid administered approximately 5 min after administration of the α_2_ agonist. All animals were induced with ketamine (Anesketin; Dechra Veterinary Products) (2.2–2.7 mg/kg bwt iv) and midazolam (Dormazolam; Dechra Veterinary Products) (0.05–0.07 mg/kg iv). The trachea was intubated and supplemental oxygen was supplied via a flowmeter (5–15 L/min). A preservative‐free, paraffin‐based bland ophthalmic ointment (Lacri‐Lube; Allergan) was applied to the ocular surface. Anaesthesia was maintained by one of three methods, first, by intravenous infusion of a mixture of guaifenesin (Myorelax; Dechra Veterinary Products) (100 mg/mL), ketamine (Anesketin; Dechra Veterinary Products) (2 mg/mL) and xylazine (Chanazine; Chanelle Pharma) (1 mg/mL) with the resultant mixture administered at 1–2 mL/kg/h. Second, via top‐up boluses of ketamine (Anesketin; Dechra Veterinary Products) (0.6–0.9 mg/kg bwt iv every 8–12 min) with romifidine (Sedivet; Boehringer Ingelheim Animal Health UK Ltd) (0.01–0.02 mg/kg bwt iv every 8–20 min) or thirdly, via inhalation of isoflurane (IsoFlo; Zoetis) in 100% oxygen. Monitoring was undertaken, including a minimum heart rate, respiratory rate, mucous membrane colour and capillary refill time and subjective monitoring of anaesthetic depth for the horses maintained intravenously, whereas horses maintained on volatile agents were monitored via a multiparameter monitor (Carescape B650; GE HealthCare) including electrocardiography, pulse oximetry and capnography.

At Hospital 2, horses were sedated with romifidine (Sedivet; Boehringer Ingelheim Animal Health UK Ltd) (0.07–0.1 mg/kg bwt iv), followed by induction with midazolam (Dormazolam; Dechra Veterinary Products) (0.03–0.1 mg/kg bwt iv) and ketamine (Anesketin; Dechra Veterinary Products) (1.5–3.5 mg/kg bwt iv) and maintained with continuous intravenous infusion of ketamine (Anesketin; Dechra Veterinary Products) (100 mg/mL), detomidine (Domidine; Dechra Veterinary Products) (10 mg/mL) and guaifenesin (Gujatal; Dechra Veterinary Products) (100 mg/mL). The average infusion rate of the mixture used to maintain anaesthesia was 1.3 mL/kg/h (0.9–2.0 mL/kg/h). Anaesthesia monitoring was undertaken including a minimum heart rate, respiratory rate and subjective monitoring of anaesthetic depth.

During the CT examination, anaesthesia was monitored by a board‐certified (ECVAA) anaesthetist at Hospital 1 and by a veterinarian experienced in anaesthesia at Hospital 2. All horses at both hospitals received a single dose of flunixin meglumine (1.1 mg/kg bwt iv) either with premedication or immediately following recovery. At Hospital 1, all recoveries from GA were either free or in the smaller horses (weighing under 250 kg) by manual restraint/assistance. At Hospital 2, all horses had rope‐assisted recoveries from GA.

#### 2.2.2 Scanning procedure

A helical *16 slice Aquilion large bore* (Canon Medical Systems; Zoetermeer) with a gantry diameter opening of 90 cm and a scanning field diameter of 70 cm was used for Hospital 1 and a helical *16 slice Philips Brilliance CT Big Bore* (Philips Healthcare) with a gantry diameter opening of 85 cm and scanning field diameter of 70 cm for Hospital 2.

At both hospitals, horses were placed in dorsal recumbency with the hindlimbs extended through the gantry. Horses were safely secured to the CT table to allow personnel to leave the room briefly during image acquisition.

**FIGURE 1:**
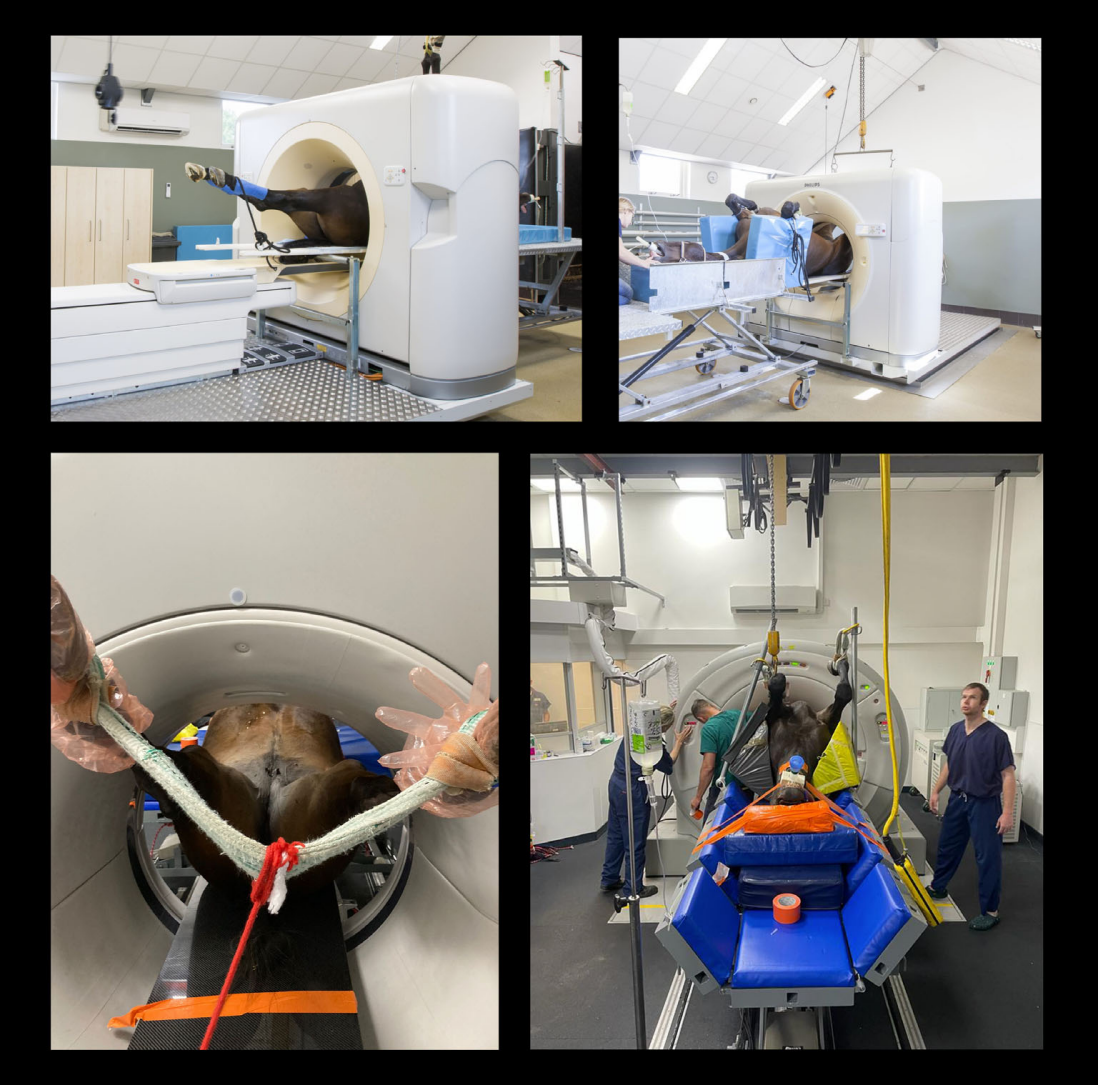
Pictures of positioning for CT acquisition at Hospital 1 (lower left and lower right) and Hospital 2 (upper left and upper right).

At Hospital 1, the horse was hoisted, using an overhead hoist, onto the table into dorsal recumbency with the rump adjacent to the CT gantry. The hoist was disconnected from the hindlimbs, which were flexed and passed caudally through the CT gantry. Once through the gantry, the hindlimbs were extended and secured in place using ropes (Figure 1; Video S1). The horse's body was maintained in position by leaving the forelimbs secured to the overhead hoist or securing the forelimbs to the table (Figure 1; Video S1). The gantry table was not padded, but the Shank's table (Hospital 1) and the custom‐built rectangular table (Hospital 2) were padded (Figure 1; Video S1). The CT examination proceeded from caudal to cranial (until the trunk touched the gantry, causing an automatic stop or manually stopped when the area of interest had been scanned) at Hospital 1. At Hospital 2, the horse was positioned within the gantry so that the trunk was touching the gantry or just cranial to the area of interest and the CT examination proceeded from cranial to caudal. An initial acquisition of a helical conventional CT scanogram was not performed and automatic exposure control (AEC) was not used (beam attenuation exceeded the ability of the AEC). Technical parameters for Hospital 1: 120–135 kVp, 115–400 mA, 0.5–1 mm slice thickness, scan FOV 40–70 cm, tube rotation time 0.75 s, and gantry pitch 0.688. Technical parameters for Hospital 2: 140 kVp, 425 mA, 2 mm slice thickness, scan FOV 60–70 cm, tube rotation time 1 s, and gantry pitch 0.938. All CT images were reconstructed using a standard or sharp bone algorithm and a smooth, soft tissue algorithm.

At Hospital 1, the sliding gantry CT machine was positioned on runners with a travel distance of 1.5 M. Automatic motors advanced the sliding gantry during scanning. A custom carbon fibre (5 × 40 × 200 cm) reinforced table was positioned through the bore and its wheels were locked in place. A surgical table (Shanks Medical) was then positioned longitudinally tightly along the base of the carbon fibre table. The side leaves of the Shank's table were raised to support the horse's shoulders and additional wedge‐shaped pads were added to ensure support. The surgical table position was locked by raising its wheels so the table rested on the axles. The anaesthetised horse, positioned and secured to the table, remained in place, while the gantry moved during scanning.

At Hospital 2, the CT machine was set up to permit the scanning of small animals from the front side and horses from the rear of the machine. The CT scanner and sliding proprietary patient table were positioned on a custom steel platform that could be moved forward and backward to facilitate the sliding of the gantry. This platform is moving during scanning. A second carbon fibre (5 × 40 × 300 cm) table was positioned through the gantry and was supported on either side of the CT bore by steel rollers positioned above the proprietary table and were affixed to the steel platform. This carbon fibre table was connected to a custom‐made rectangular table with removable bolts. The custom table was on hard rubber wheels on a hard rubber‐coated floor to provide smooth movements and was locked so that only forward/backwards motion was possible. The pelvis was on the carbon fibre table, while the thorax, cervical spine and head were lying on a polyurethane bed with supporting pads beside the thorax. The anaesthetised horse, positioned and secured to the table, remained in place, while the gantry, together with the steel platform, moved during scanning.

#### 2.2.3 CT imaging evaluation

Images were retrieved and graded simultaneously by a board‐certified (ACVSMR) equine sports medicine and rehabilitation veterinarian (N.K.O) and board‐certified (ECVS) equine surgeon (J.D.S) (both experienced in CT reading) on a computer monitor using proprietary DICOM software in single and multiplanar views (Horos v3.3.6 DICOM viewer). Reviewers were not blinded to the clinical information and previous imaging reports were available to reviewers at the time of image analysis. If the reviewers disagreed on grading, relevant images were carefully considered and compared with the grading standard until a consensus was reached. Any structures that were unable to be assessed due to the FOV or artefact/image quality were excluded from grading.

#### 2.2.4 Image quality

**FIGURE 2:**
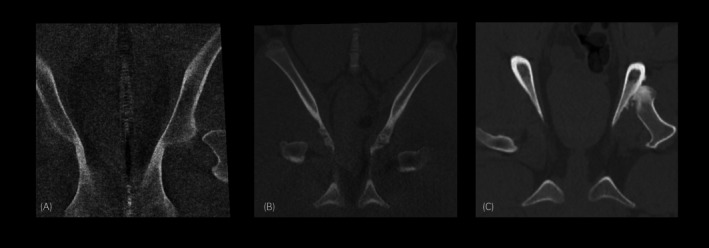
Examples of CT images grades as ‘Poor or suboptional study; images were diagnostic but were noisy (1)’ (A), ‘Acceptable study; images were diagnostic but slightly noisy (2)’ (B) and ‘Good or excellent study; diagnostic with minimal noise not affecting image quality (3)’ (C).

The diagnostic quality of the CT was graded as ‘Nondiagnostic study; images had an unacceptable level of noise preventing image interpretation (0)’, ‘Poor or suboptional study; images were diagnostic but were noisy (1)’, ‘Acceptable study; images were diagnostic but slightly noisy (2)’ and ‘Good or excellent study; diagnostic with minimal noise not affecting image quality (3)’ (Figure 2). Image artefacts were recorded for each case. Images were viewed in all three planes (dorsal, transverse and sagittal) and aligned to the anatomical region being assessed. Windowing was adjusted to maximise the quality (contrast and brightness) of the images. Maximum intensity projection images were also used during image interpretation and parameters were altered accordingly.

#### 2.2.5 Field of view

The FOV consisting of the most cranial, caudal and lateral anatomical areas included in the examination were recorded. For horses that underwent more than one CT study of the caudal spine/pelvis, the cranial extent of the cranial study and the caudal extent of the caudal study were recorded. The number of individual CT studies performed under the same GA was recorded.

### 2.3 Anatomical description

Each study was assessed systematically and the anatomy, anatomical variations, congenital, developmental and pathological features were described by the reviewers (ACVSMR and ECVS). Pathological findings of the sacroiliac joint, pelvis and coxofemoral joints are reported in more detail in a separate article^22^ and cases are summarised in Table S1.

The number of lumbar vertebrae was recorded. The inclination (‘caudal’, ‘cranial’ or ‘straight’) of the dorsal spinous processes and transverse processes of the lumbar spine was recorded. The presence of impingement (‘present’ or ‘absent’) between dorsal spinous processes and between transverse processes was also noted. The location of the diverging interspinous space in the caudal lumbar spine or lumbosacral space was recorded (‘L5‐6’, ‘L6‐S1’ or ‘L5‐6 and L6‐S1’). The intertransverse joints in the lumbar spine were recorded as ‘present’ or ‘absent’. The degree of fusion (‘fused’, ‘partially fused’ or ‘not fused’) and modelling (‘no sclerosis/irregular new bone’ or with ‘sclerosis/irregular new bone’) at the intertransverse joints and lumbar and lumbosacral articular process joints were graded (Table 1). The transverse processes of the most caudal lumbar vertebra were categorised as ‘lumbar‐like’ or ‘sacral‐like’ if the shape mimicked the typical shape of the transverse processes of lumbar or sacral vertebrae (Figure S1).^23^

**TABLE 1:** Grading for articular process joints and intertransverse joints.

Grade 0	Grade 1	Grade 2	Grade 3	Grade 4	Grade 5	Grade 6
N/a (not in FOV or unable to assess due to artefact/image quality)	Completely fused without new bone formation or sclerosis	Completely fused with new bone formation and/or sclerosis	Partially fused without new bone formation or sclerosis	Partially fused with new bone formation and/or sclerosis	Not fused without new bone formation or sclerosis	Not fused with new bone formation and/or sclerosis

The dorsal spinous processes of the lumbar and sacral vertebrae were assessed for spina bifida. Spina bifida was defined as a dorsal bony defect at midline or just off midline. Spina bifida was recorded as ‘present’ or ‘absent’.

The number of sacral vertebrae was counted when the most cranial aspect and caudal aspect of the sacrum were included in the FOV; when either the cranial or caudal border was outside of the FOV, the number of vertebrae was not able to be counted. The number of sacral vertebrae and fusion between the sacral dorsal spinous processes (‘ventral’, ‘dorsal’, ‘ventral and dorsal’, ‘central’ or ‘not fused’) of the sacral vertebrae were recorded. The amount of fusion was calculated by measuring the height of the dorsal spinous processes; measured from the most dorsal aspect of the spinal canal to the dorsal summit of the spinous process. A second measure, the fused region, was then obtained and a percentage was calculated. If the dorsal summit of the dorsal spinous process or the dorsal aspect of the vertebral canal was outside of the FOV, the percentage was not calculated.

### 2.4 Data analysis

Data were imported from Excel into SPSS and assessed for normality using Shapiro–Wilk tests. Normally distributed data were presented as mean and standard deviation, and non‐normally distributed data were presented as the median and interquartile range (IQR). Quantitative data with two groups were compared using Mann–Whitney *U* tests and with more than two groups using Kruskal–Wallis tests. Association between categorical data was compared using chi‐square test, or when more than 20% of cells had expected frequencies <5 Fisher's exact test was used instead. Correlation between bodyweight and GA time, and ‘fusion’, ‘partial fusion’ and ‘no fusion’ between the caudal intertransverse joints and articular process joints of the lumbar spine was assessed using Spearman's test for correlation. Confidence intervals (CIs) were calculated for proportions using Exact (Binominal) Symmetrical 95% CI. Significance was set to 0.05.

## 3 RESULTS

### 3.1 Signalment

Sixty‐five horses with CT studies of the pelvis/caudal spine were retrieved out of which 56 horses met the inclusion criteria and were included in the study (Figure S2). Four horses did not meet the inclusion criteria based on age (under 6 months) and five horses based on the CT being performed *post‐mortem*. Out of the 56 horses included in the study, 13 horses were from Hospital 1 and 43 horses were from Hospital 2.

Ages ranged from 8 months to 20 years (mean: 8.4 years, median: 7 years), with no statistical difference between the two hospitals. The bodyweights were between 85 and 680 kg (mean: 488 kg, median: 528 kg).

Warmblood or warmblood crosses (*n* = 28) were most common. Other horse breeds included Friesian (*n* = 6), Thoroughbred (*n* = 3), cob (*n* = 2), Appaloosa (*n* = 2), Standardbred (*n* = 1), Andalusian (*n* = 1), Irish Hunter (*n* = 1) and Arabian (*n* = 1). Pony breeds included Miniature horse (*n* = 2), Welsh Sections A and B (*n* = 2), Icelandic horse (*n* = 2), Shetland pony (*n* = 2), New Forest pony (*n* = 1), unknown pony breed (*n* = 1), and Highland pony (*n* = 1). Male horses (37/56), geldings (30/56) and colts/stallions (7/56) were more prevalent than mares/fillies (19/56).

### 3.2 Anaesthesia

Anaesthetic records were available for all 56 horses. No adverse events were recorded, and all horses recovered, without incident, from GA.

Anaesthesia time ranged from 10 to 65 min (median: 32 min, IQR: 20 min). The median for Hospital 1 was 30 min (IQR: 12.5 min) and the median for Hospital 2 was 30 min (IQR: 20 min) with no statistical difference between the anaesthesia time between the two hospitals (*p* = 0.4). There was no statistically significant correlation between bodyweight and GA time (*p* = 0.7).

The mean GA time for horses that had one CT study was 29 min (median: 30 min, IQR: 28 min), for horses that had two CT studies the mean GA time was 36 min (median: 30 min, IQR: 20 min) and for horses that had three CT studies the mean GA time was 39 min (median: 30 min, IQR: 19 min), with no statistical difference between the three categories (*p* = 0.09).

### 3.3 CT imaging evaluation

#### 3.3.1 Field of view

The FOV varied between cases based on the area of interest for each clinical case and the horse's size. The cranial extent of the FOV was limited by the widest part of horse's trunk contacting the CT gantry. Additional CT studies of other regions under the same GA included the cervical spine, stifle, tarsus and distal limb. Additional CT scans of the caudal spine and/or pelvis were performed to extend the cranial/caudal FOV.

In both hospitals, most horses (29/56; 51.7%, CI: 38.0%–65.3%) had a single CT study of the caudal spine and/or pelvis (8 horses at Hospital 1 and 21 at Hospital 2). Horses that had two CT studies (21/56, 37.5%, CI: 24.9%–51.5%) either had two scans of the caudal spine and/or pelvis to increase the FOV (12 horses), or one scan of the pelvis/caudal spine and a separate scan of a different region (9 horses: stifles [*n* = 7], tarsus [*n* = 1] and distal limb [*n* = 1]). Only a small number of horses had three CT studies (6/56, 10.7%, CI: 4.0%–21.9%). Two horses had three separate studies of the caudal spine and/or pelvis to increase the FOV, and four horses had three separate studies where one/two studies were of the caudal spine and/or pelvis and one/two separate scans of different regions (stifles [*n* = 2], cervical spine [*n* = 1], tarsus [*n* = 1] and distal limb [*n* = 1]).

The cranial extent of the scan varied between the 16th thoracic vertebra to the 5th sacral vertebra, with most cases having the cranial extent of the scan at the caudal lumbar spine (Figure S3). The caudal extent of the scan varied from the first sacral vertebra to include all of the rump of the horse (Figure S3). All the sacrum was included in the FOV in most horses (45/56, 80.4%, CI: 67.6%–89.8%), and all of the rump was included in the FOV in a small number of horses (6/56, 10.7%, CI: 4.0%–21.9%). All of the lateral osseous and soft tissues were included in almost a third of cases (16/56, 28.6%, CI: 17.3%–42.2% on the right, and 17/56, 30.4%, CI: 18.8%–44.1% on the left). All or most of the *tuber coxae* were included in most of the cases (44/56, 78.6%, CI: 65.6%–88.4% on the right and 43/56, 76.8%, CI: 63.6%–87.0% on the left) (Figure S3).

#### 3.3.2 Diagnostic quality grade

**FIGURE 3:**
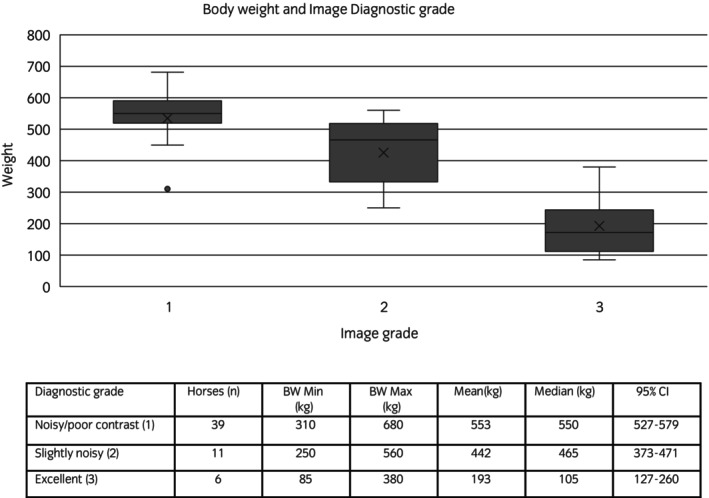
Box and Whiskers plot demonstrating the image grade and weight association.

The image quality grade was considered diagnostic in all cases, ranging between 1 and 3 (median: 1, IQR: 1). There was a higher diagnostic grade for CT scans obtained at Hospital 1 (min: 2, max: 3, median: 2, IQR: 1) compared with those achieved in Hospital 2 (min: 1, max: 2, median: 1, IQR: 0) (*p* < 0.01). The image quality was better for horses with lower bodyweights than for horses with higher bodyweights (*p* < 0.01) (Figure 3). There was a statistically significant lower bodyweight for horses at Hospital 1 (min: 85 kg, max: 526 kg, median: 350 kg, IQR: 231 kg) than horses at Hospital 2 (min: 285 kg, max: 680 kg, median: 540 kg, IQR: 100 kg) (*p* < 0.001).

Image artefacts were common at both hospitals (Figure S4). A degree of noise (quantum mottle) was present in areas with a large cross‐sectional area, particularly in large horses. At Hospital 2, all scans had ring artefacts present, seen as concentric, alternating hypo‐ and hyperdense rings emanating from the isocentre of the scan. Poisson noise, seen as bright and dark streaks that appear preferentially along the direction of greatest attenuation, was most common in scans from Hospital 2. At both hospitals, there was beam hardening present at the cranial and caudal cortices of the proximal femur, the *os ischium* and *pubis*, and to a lesser extent the ilial shaft. As the streaking occurred caudal to the sacroiliac region interpretation of this area was not affected. In addition, beam hardening caused by reinforcing carbon fibre support beams on the underside of the table was seen at Hospital 1.

### 3.4 Anatomical variations

#### 3.4.1 Lumbar intertransverse joints

The intertransverse joint findings have been summarised in Figure S5. Intertransverse joints were more commonly present in the caudal lumbar spine; however, in a small number of horses intertransverse joints were present within the cranial lumbar spine as well. Intertransverse joints were not present in any of the seven horses with scans of L1‐2. One horse, out of the 14 horses with scans of this region, had bilateral intertransverse joints at L2‐3 (1/14, 7.1%, CI: 0.2%–33.9%). The intertransverse joint at L3‐4 was assessed in 21 horses with intertransverse joints present in two horses at this site (2/21, 9.5%, CI: 1.2%–30.4%). The intertransverse joint at L4‐5 was assessed in 30 horses, with an intertransverse joint present in over half horses (19/30, 63.3%, CI: 43.9%–80.1%). The intertransverse joints at L5‐6 and L6‐S1 were assessed in 43 and 49 horses, respectively, with intertransverse joints present at both these sites in all horses (CI: 91.8%–100.0% and 92.8%–100.0%, respectively).

Partial or complete fusion (unilateral or bilateral) was not present at the intertransverse joints in the cranial lumbar spine (L2‐3 and L3‐4) (in any of the 14 and 21 horses where this region was assessed, respectively). Partial or complete fusion of the intertransverse joints was less common at L4‐5 (8/19, 42.1%, CI: 20.3%–66.5%) than at L5‐6 (24/43, 55.8%, CI: 39.9%–70.9%). Partial or complete fusion of the intertransverse joints was uncommon at L6‐S1 (9/49, 18.4%, CI: 8.8%–32.0%).

At L4‐5 there was a bilateral complete fusion of the intertransverse joints in two horses (with no sclerosis or new bone formation) (2/19, 10.5%, CI: 0.1%–33.1%). Partial fusion was present bilaterally in six horses (6/19, 31.6%, CI: 12.6%–56.6%), with three having sclerosis and/or new bone formation and three having no sclerosis and/or new bone formation. There was no fusion in the remaining 11 horses (11/19; 57.9%, CI: 33.5%–79.8%) (however, five of these horses had sclerosis and/or new bone formation).

At L5‐6, the intertransverse joints were completely fused bilaterally in 10 horses (10/43, 23.3%, CI: 11.8%–38.6%), with one horse having fusion with sclerosis and/or new bone formation and the remaining nine horses having fusion without sclerosis and/or new bone formation. There was a bilateral partial fusion in 12 horses (12/43, 27.9%, CI: 15.3%–43.7%), with nine horses having partial fusion with sclerosis and/or new bone formation and the remaining three horses having partial fusion without sclerosis and/or new bone formation. Two horses (2/43, 4.7%, CI: 0.6%–15.8%) had partial unilateral fusion and complete fusion on the contralateral side, with no sclerosis and/or new bone formation on either side. There was no fusion in the remaining 19 horses (19/43, 44.2%, CI: 29.1%–60.1%) (only 3 of these horses had sclerosis and/or new bone formation).

There was no fusion of the intertransverse joints at L6‐S1 in most horses (40/49, 81.6%, CI: 68.0%–91.2%). Out of the joints that were not fused, there was no sclerosis and/or new bone formation bilaterally in 37 of these horses and sclerosis and/or new bone formation was present bilaterally in two horses. In one horse, without fusion, the joint had unilaterally no sclerosis or new bone formation and on the contralateral side sclerosis and/or new bone formation.

**FIGURE 4:**
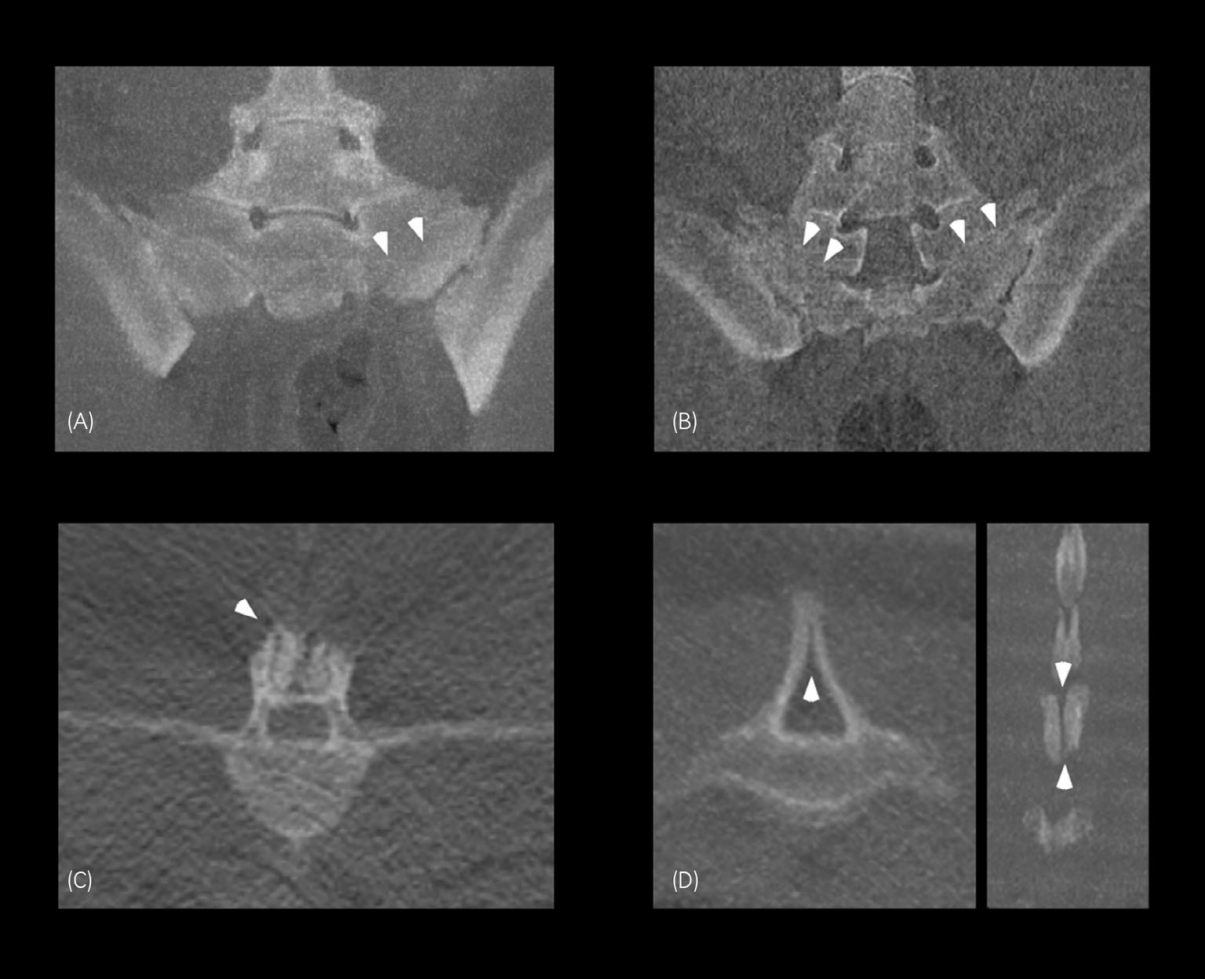
CT images (in bone window). Left is to the right in transverse and frontal images and cranial is to the left in sagittal images. Transverse (A) and frontal (B) images centred at the sacroiliac joint demonstrating unilateral fusion (A) and bilateral fusion (B) of the L6 and sacrum (white arrows). A transverse image (C) centred over lumbar articular process joints demonstrating asymmetry between the left and right side and new bone formation and enlargement of the left articular process joint (white arrowhead). A transverse (left) and sagittal (right) image and (D) centred over the sacrum showing multiple sites of spinal bifida (white arrows).

The intertransverse joints at L6‐S1 were bilaterally completely fused (sacralisation) in a small number of horses (4/49, 8.2%, CI: 2.3%–19.6%), all with no sclerosis and/or new bone formation present. Two horses (2/49, 4.1%, CI: 0.5%–14.0%) had a bilateral partial fusion, with sclerosis and/or new bone formation. Two horses (2/49, 4.1%, CI: 0.5%–14.0%) had unilateral fusion without sclerosis and partial fusion of the contralateral side (one horse had sclerosis/new bone formation on the partial fused side and one had no sclerosis/new bone formation on the partial fused side) (Figure 4) and one horse (1/49, 2.0%, CI: 0.1%–10.9%) had a joint that was partially fused unilaterally and no fusion on the contralateral side, both sided without sclerosis or new bone formation.

There was no association with age and grade of fusion/modelling (Grades 1–6; Table 1) at any site of the lumbar or lumbosacral intertransverse joints (*p* = 0.2). There was no association between age and fusion at any site of the assessed lumbar spine when grouped into ‘fused’ (Grades 1 and 2), ‘partially fused’ (Grades 3 and 4) and ‘not fused’ (Grades 5 and 6) (*p* = 0.5) or when grouped based on ‘new bone formation and/or sclerosis’ or ‘no new bone formation/or sclerosis’ (Groups 1, 3 and 5 and Groups 2, 4 and 6) (*p* = 0.3).

#### 3.4.2 Lumbar transverse processes

The most common inclination of the transverse processes was straight in the cranial lumbar spine (L1‐3), with three out of six (3/6, 50.0%, CI: 11.8%–88.2%) horses having a straight inclination at L1, and all horses at L2 (11/11, 100.0%, CI: 71.5%–100.0%) and L3 (15/15, 100.0%, CI: 78.2%–100.0%) having a straight inclination at L3. In the caudal lumbar spine, a cranial inclination was more common (18/27, 66.7%, CI: 46.0%–83.5% at L4, 36/39, 92.3%, CI: 79.1%–98.4% at L5 and 41/44, 93.2%, CI: 81.3%–98.6% at L6) (Figure S6). The inclination of the transverse processes was the same on the left and right sides in all but one horse, where the transverse process of L3 on the right side was caudal and the alignment on the left side was straight (this horse was counted both as straight inclination and caudal inclination above).

Impingement of the transverse processes was most common in the mid‐lumbar spine (L3‐4 and L4‐5) (Figure S7). At L3‐4, bilateral impingement was present in six horses (6/19, 31.6%, CI: 12.6%–56.6%). At L4‐5, bilateral impingement was present in 12 horses (12/27, 44.4%, CI: 25.5%–64.7%). At L5‐6, bilateral impingement was present in two horses (2/39, 5.1%, CI: 0.6%–17.3%). At L6‐S1, bilateral impingement was present in one horse (1/45, 2.2%, CI: 0.1%–11.8%). In most horses, impingement was present bilaterally when present, however unilateral impingement was present in two horses (2/13, 15.4, CI: 1.9%–45.5%) at L2‐3 and in one horse (1/27, 3.7%, CI: 0.1%–19.0%) at L4‐5. Complete fusion between the transverse processes was present in one horse (1/39, 2.6%, CI: 0.1%–13.5%) bilaterally at L5‐6, and unilaterally in two horses (2/45, 4.4%, CI: 0.5%–15.2%) at L6‐S1.

#### 3.4.3 Shape of the last lumbar vertebra

The shape of the last lumbar vertebra was assessed in 44 horses. There was a similar prevalence of ‘lumbar‐shape’ and ‘sacral‐shape’ of the last lumbar vertebra with 21 horses having a ‘lumbar‐shaped’ L6 (21/44, 47.7%, CI: 32.5%–63.3%) and 23 horses having a ‘sacral‐shaped’ L6 (23/44, 52.3%, CI: 36.7%–67.5%). In one horse, the last lumbar vertebra was asymmetric between left and right, with the right side being ‘sacral‐shaped’ and the left side being ‘lumbar‐shaped’. Warmblood and Warmblood crosses more commonly had a ‘lumbar‐shaped’ L6 (13/21, 61.9%, CI: 38.4–81.9) than a ‘sacral‐shaped’ L6.

The shape of L6 was not statistically associated with an imaging (based on CT) diagnosis of sacroiliac joint osteoarthritis (*p* = 0.8), intervertebral disc disease (*p* = 0.4) or ventral spondylosis (*p* = 0.7).

#### 3.4.4 Fusion and modelling of the lumbar articular process joints

The articular process joint findings have been summarised in Figure S5. The articular process joints were assessed in eight horses at L1‐2. At this site, there was no fusion, sclerosis and/or new bone formation in any of the joints.

The articular process joints were assessed in 16 horses at L2‐3. There was no fusion or sclerosis and/or new bone formation in the majority of joints at this site (13/16, 81.3%, CI: 54.6%–96.0%). In one horse (1/16, 6.3%, 0.2%–30.2%), the joints were not fused; however, there was sclerosis and/or new bone formation. In two horses (2/16, 12.5%, 1.6%–38.4%), the joints were partially fused bilaterally, with one horse having no sclerosis and/or new bone formation and one horse having sclerosis and/or new bone formation.

The articular process joints were assessed in 23 horses at L3‐4 with no fusion present in any of these joints, and there was no sclerosis and/or new bone formation. The articular process joints were assessed in 32 horses at L4‐5. There was no fusion bilaterally in almost half of these horses (14/32, 43.8%, 26.4%–62.3%). In one horse without fusion, there was sclerosis and/or new bone formation present bilaterally. Partial or complete fusion was present in the remainder of the horses (18/32, 56.3%, 27.7%–73.6%). Partial fusion was present bilaterally in four horses, with two having sclerosis and/or new bone formation and two horses having no sclerosis and/or new bone formation. Complete fusion was present bilaterally in 13 horses, with 11 horses having fused joints with no sclerosis and/or new bone formation and two horses having sclerosis and/or new bone formation.

The articular process joints were assessed in 43 horses at L5‐6. There was no fusion present in 16 horses (16/43, 37.2%, CI: 23.0%–53.3%). Out of these horses, four had sclerosis and/or new bone formation. Partial or complete fusion was present in the remaining 27 horses (27/43, 62.8%, CI: 46.7%–77.0%). Partial fusion was present bilaterally in 13 horses, with six horses having sclerosis and/or new bone formation present bilaterally. Complete fusion was present in 14 horses, 11 of these had bilateral fusion without sclerosis and/or new bone formation and 2 horses had bilateral sclerosis and/or new bone formation. One horse had complete fusion without sclerosis and/or new bone formation on the right side and no sclerosis and/or bone formation on the left side.

The articular process joints were assessed in 47 horses at L6‐S1. There was no fusion bilaterally in most of these horses (35/47, 74.5%, 56.7%–86.1%). In two horses without fusion, there was sclerosis and/or new bone formation present bilaterally. Partial or complete fusion was present in the remainder of the horses (12/47, 25.5%, 14.0%–40.4%). Bilateral partial fusion was present in four horses, with two having sclerosis and/or new bone formation and two horses having no sclerosis and/or new bone formation. One horse had partial fusion on one side and complete fusion on the other side, with neither side having new bone formation or sclerosis. Complete fusion was present bilaterally in seven horses, with five horses having fused joints with no sclerosis and/or new bone formation and two horses having sclerosis and/or new bone formation.

There was no association with age and grade of fusion at any site of the lumbar articular process joints (*p* = 0.3). There was no association with age when horses were grouped into ‘fused’ (Grades 1 and 2), ‘partially fused’ (Grades 3 and 4) and ‘not fused’ (Grades 5 and 6) (Table 1) (*p* = 0.5), or when grouped based on ‘new bone formation and/or sclerosis’ or ‘no new bone formation and/or sclerosis’ (Groups 1, 3, and 5 and Groups 2, 4 and 6) (*p* = 0.1).

There was a strong correlation between the fusion of the articular process joints and intertransverse joints at L6‐S1 (*r* = 0.7, *p* < 0.001) and a moderate correlation of fusion at the articular process joints and intertransverse joints at L5‐6 (*r* = 0.4, *p* = 0.05).

#### 3.4.5 Dorsal spinous process inclination and impingement in the lumbar spine

The dorsal spinous processes were assessed at L1 in 6 horses, L2 in 11 horses, L3 in 18 horses, L4 in 31 horses, L5 in 43 horses and L6 in 47 horses (Figures S8 and S9). A cranial or straight inclination of the dorsal spinous processes was most common in the lumbar spine; at L1, half of the horses had a straight inclination and half had a cranial inclination (3/6, 50.0, CI: 11.8%–88.2%, for both), at L2, 7 horses had a cranial inclination and four horses a straight inclination (7/11, 63.6, CI: 30.8%–89.1% and 4/11, 36.4%, CI: 10.9%–69.2%, respectively), at L3, 11 horses had a cranial inclination and 7 horses a straight inclination (11/18, 61.1%, CI: 35.8%–82.7% and 7/18, 38.9%, CI: 17.3%–64.3%, respectively), at L4, 24 horses had a cranial inclination and 4 horses had a straight inclination (24/29, 82.8%, 64.2%–94.2% and 4/29, 13.8%, CI: 3.9%–31.7%, respectively), at L5, 38 horses had a cranial inclination (38/43, 88.4%, CI: 74.9%–96.1%) and at L6, 22 horses had a cranial inclination and 19 horses had a straight inclination (22/47, 46.8%, CI: 32.1%–61.9% and 19/47, 40.4%, CI: 26.4%–55.7%, respectively). A caudal inclination was only present in a small number of the three most caudal lumbar dorsal spinous processes (L4, L5, L6) (L4 1/29, 3.5%, CI: 0.1%–17.8%, L5 5/43, 11.6%, CI: 3.9%–25.1%, L6 6/47, 12.8%, CI: 4.8%–25.7%).

The divergent (widest) interspinous space of the lumbosacral spine was most common at L6‐S1 (30/47, 63.8%, CI: 48.5%–77.3%), followed by L5‐6 (12/47, 25.5%, CI: 14.0%–40.4%). In a small number of horses, there was a similar degree of divergence at both these locations (5/47, 10.6%, CI: 3.6%–23.1%).

There was no statistically significant association between the location of the divergent (widest) interspinous space and an imaging diagnosis of sacroiliac joint osteoarthritis (*p* = 0.2) or ventral spondylosis (*p* = 0.3) (based on CT). There was a statistically significant association between the location for the divergence (widest) interspinous space and the horse being diagnosed (based on CT) with intervertebral disc disease (*p* = 0.03), with a higher than expected number of horses having a diagnosis of intervertebral disc disease of the caudal lumbar/lumbosacral spine when the divergence was located at both ‘L6‐S1’ and ‘L5‐6’ and when the divergence was located at ‘L5‐6’, and a lower than expected number being diagnosed with intervertebral disc disease when the divergence was located at ‘L6‐S1’.

Impingement of the lumbar dorsal spinous processes was only found at L4‐5 in this study, and only in seven horses (Figure S9).

#### 3.4.6 Spina bifida

Spina bifida in the lumbosacral spine was present in nine horses (Figure 4). Spina bifida was present in two horses in the lumbar spine; one horse had multilevel spina bifida with two adjacent vertebrae affected (L6 and L7) and in one horse, L6 alone was affected. Spina bifida was present in the sacral spine in seven horses. Four of these horses had multilevel spina bifida; two horses having three adjacent vertebrae affected (S3, S4 and S5) and two horses having two vertebrae affected (S3 and S4 and S3 and S5, respectively). Three horses had one vertebra affected at S4, S5 and S6, respectively.

In eight horses the spina bifida were closed and seen as small midline defects in the dorsal spinous process. One horse had a chronic (years) history of a draining tract over the caudodorsal aspect of the sacrum with no history of trauma. CT revealed a gas‐filled tract extending from the dorsal skin, extending ventrally and cranially, entering the spinal canal between the last sacral vertebra and the first coccygeal vertebra, compatible with a dorsal dermal sinus. A midline defect was present in the last sacral dorsal spinous process.

There was no statistically significant association between horses having spina bifida and an imaging diagnosis of intervertebral disc disease (*p* > 0.9), sacroiliac joint osteoarthritis (*p* > 0.9), or ventral spondylosis (*p* > 0.9) (based on CT).

#### 3.4.7 Sacrum

The number of sacral vertebrae was able to be counted in 49 horses (both the cranial and caudal most borders of the sacrum were included in the FOV), with the number of sacral vertebrae varying between four to six (Figure S10; Table S1). Most horses (35/49, 71.4%, CI: 56.7%–83.4%) had five sacral vertebrae. Only 1 horse had four sacral vertebrae (1/49, 2.0%, CI: 0.1%–10.9%) and the remaining 13 horses had six sacral vertebrae (13/49, 26.5%, CI: 15.0%–41.1%).

The sacral dorsal spinous processes fusion was able to be calculated in 53 horses at S1‐2 and S2‐3, in 52 horses at S3‐4, in 51 horses at S4‐5 and in 13 horses at S5‐6 (Figure S5). No fusion or ventral fusion was most common at all sites. No fusion was most common at S1‐2 (41/53, 77.4%, CI: 63.8%–87.7%), followed by S2‐3 (26/53, 49.1%, CI: 35.1%–63.2%), S3‐4 (19/53, 36.5%, CI: 23.6%–51.0%), S4‐5 (41/51, 80.4%, CI: 66.9%–90.2%) and S5‐6 (13/13, 100.0%, CI: 75.3%–100.0%). Ventral fusion was most common at S3‐4 (30/52, 57.7%, CI: 43.2%–71.3%). Both central fusion and combined dorsal and ventral fusion were uncommon in all sites.

## 4 DISCUSSION

This is the first study to describe CT imaging of the caudal spine and pelvis in clinical cases, including full‐size adult horses. We have outlined the techniques and technological advances in terms of sliding gantries and novel modifications to infrastructure around the CT gantry, including patient tables, that allowed CT imaging of the equine pelvis and caudal spine under GA. We have described the protocols and processes to acquire diagnostic scans using two different CT machines in two equine hospitals and the anatomical variations observed in these clinical cases.

The main advantage of CT imaging over other imaging modalities is the ability to produce cross‐sectional images without superimposition, permitting assessment of all aspects of multiple anatomical structures within the scanned region.^17^, ^24^ However, due to the increased mass of the equine pelvis, the image quality was lower than that typically obtained from the equine cervical region. This limited the diagnostic capability in some areas.

None of the horses in this study sustained any injury or disease due to their procedures despite the variations in the anaesthetic protocols and the positioning required. This may be in part due to the brevity of anaesthesia.^25^ The position of the hindlimbs necessary for CT scanning has been reported to be a risk factor for femoral nerve paralysis. The position should only be maintained for as long as necessary to obtain the scans and extended periods of bilateral hindlimb extension should be avoided.^26^, ^27^ All horses received a single dose of flunixin meglumine, which may have masked mild muscle soreness but was unlikely to mask significant myopathy/neuropathy. Pre‐anaesthetic NSAIDs have been used in horses undergoing elective MRIs under GA.^26^ The use of pre‐anaesthetic NSAID was used routinely for all horses in this study to reduce possible pain associated with manipulation under GA, particularly in horses with painful disorders such as fractures and/or osteoarthritis. Within this study population, GA for CT was performed in nine horses with pelvic fractures,^22^ something that has been contraindicated historically.^28^ All but one of these fractures were chronic and none were suspected to have involved the ischial shaft. GA of horses with suspected pelvic fractures should be considered very carefully, particularly in horses with possible ischial/ilial shaft fractures as these are at particular risk for fatal haemorrhage.^28^

Horses imaged in this study weighed up to 680 kg, and as such, we have not established an upper weight limit, but it stands to reason that the maximum width of the pelvis, namely the distance from the tip of each tuber coxae, could not exceed the bore diameter. We saw a preponderance of lower‐quality images in heavier horses due to greater attenuation of the CT beam resulting in noisy images.

Several artefacts related to relative underexposure were evident on scans. Noise was the most common artefact and lowered signal‐to‐noise ratio in larger ponies and horses. Noise occurs due to photon starvation from underexposure.^16^, ^29^, ^30^ The exposure settings, most importantly mA, although set to the CT machine's maximum setting in both hospitals, resulted in relative underexposure due to the large size of the targets. High noise lowers the signal‐to‐noise ratio, decreasing the picture quality and contrast resolution.^16^, ^29^, ^30^, ^31^ In images with low signal‐to‐noise ratios, maximum intensity projections were used to aid interpretation in the viewing software.

Poisson noise and ring artefacts were other exposure‐related artefacts noted in this study.^30^ Both were more commonly encountered at Hospital 2. Ring artefact appeared close to the isocentre of the bore and was visible over multiple slices. The appearance of this artefact was consistent and resulted from the failure to deliver sufficient radiation dose.^31^ The artefact was rarely overlying the region of interest so its impact on diagnostic quality was low. Poisson noise affected the periphery of the images. There was also beam hardening in this case series, with the artefact occurring predictably in certain locations.^31^ These included the cranial and caudal cortices of the proximal femur, the *os ischium* and pubis, and to a lesser extent, the ilial shaft. As the streaking occurred caudal to the lumbosacroiliac region interpretation of this area was not affected.

Even with these issues, the images were of diagnostic quality and permitted assessment of relatively small anatomical structures such as the articular process joints. It is challenging to directly compare the FOV and image quality due to the differences in breeds and sizes between the hospitals. However, the Canon CT machine appeared to outperform the Philips machine in the quality of images, and the wider bore allowed imaging to extend more cranially. The generator power rating for the Canon machine is higher (60 kW output), with a higher maximum mA range and more kV steps, compared with the Philips (48 kW output), accounting in part for the higher resolution. The Canon machine could also scan at a smaller pitch compared with the Philips machine without overheating.

The anatomical regions that were imaged varied between cases according to the region of clinical suspicion. Scanning of the entirety of the pelvis and lumbar spine was not indicated in all cases. Scans of such large areas required CT machines to operate close to maximum exposures and required periods between CT studies to allow cooling of the CT anode. Prolonging the duration of anaesthesia to image areas outside the region of clinical interest was not undertaken on ethical grounds. This led to a somewhat heterogenous array of scan regions and limited our ability to compare scan protocols between hospitals and between horses. As not all lumbar vertebrae were imaged, we assumed these horses had six lumbar vertebrae and called the most caudal lumbar vertebra the sixth lumbar vertebrae irrespectively. When all lumbar vertebrae were imaged, we counted caudad from the last thoracic vertebrae, an approach described previously.^22^ However, an alternate nomenclature has been proposed counting cranial from the sacrum.^22^ We elected to use the established nomenclature but acknowledge that the most caudal lumbar vertebrae may not have been L6 in all instances.

A range of lumbosacroiliac anatomic variations have been described in horses. Most variations have only been detectable post‐mortem.^3^, ^22^, ^32^, ^33^, ^34^ Anatomical variations diagnosed by CT in clinical cases in this study included fusion of the lumbar transverse processes, fusion of the sacral dorsal spinous processes, presence of lumbosacral transitional vertebrae, varying number of lumbar and sacral vertebrae and variation of the location of widest interspinous divergence.

The widest interspinous divergence in this study was most prevalent between L6 and S1. In a cadaver study using CT, a similar proportion (60%) of horses also had the widest interspinous divergence at this location.^22^ However, in an earlier study by the same author where the cadavers were assessed by dissection, a larger proportion (87%) of horses had the divergent (widest) interspinous space between L6 and S1.^3^ In horses, a correlation between the dorsal spinous process orientation of the last lumbar vertebra and irregularities of the end plate of the same vertebra, as well as abnormalities of the L6‐S1 and L5‐6 symphyses has been described.^22^ Some authors have speculated that having the widest interspinous divergence between L5 and L6 may induce abnormal stresses on the lumbosacral intervertebral joint and has been related to lumbosacroiliac pathology.^35^, ^36^ In this study, an association was found between the location of the divergence and a diagnosis of interverbal disc disease of the caudal lumbar/lumbosacral spine. However, it is important to recognise that only 12 horses out of the 56 included in the study had a diagnosis of vertebral disc disease and a larger population of horses would be needed to determine the significance of this association. Furthermore, we found no association between the location of the divergence and a diagnosis of sacroiliac joint osteoarthritis.

Osteoarthritis of the articular process joint has been linked to back pain in horses,^37^, ^38^ and an association has been described between pathology of dorsal spinous processes in horses and articular process joint pathology.^39^ Radiography and ultrasonography, and recently CT of dissected lumbar specimens, have been described for assessment of the articular process joints in horses.^6^, ^37^, ^38^, ^39^, ^40^ CT (in cadavers) was found to have superior intraobserver agreement compared with ultrasonography for assessment of osteoarthritis of the articular process joints in the thoracolumbar spine in horses.^6^ CT image quality in dissected specimens is higher compared with CT of live clinical cases due to less attenuation of the CT beam and smaller scan fields. In clinical cases differentiating developmental and pathological change was more challenging.

The shape of the articular process joints changes from T16 caudally, with the joints becoming more vertical,^36^ and this change accounts for reduced axial rotation and lateral bending.^41^, ^42^, ^43^ A *post‐mortem* study found more severe changes in the articular process joints caudal to the caudal thoracic spine,^44^ while a radiographic study described osteoarthritis more commonly in the caudal thoracic and cranial lumbar spine (T15‐LI),^38^ and recently, a cadaver study using CT described the more severe changes in the caudal thoracic and cranial to mid‐lumbar spine.^6^ We found partial and complete fusion, as well as modelling of articular process joints commonly in the caudal lumbar spine, but did not image the entire thoracolumbar spine.

Contrary to previous research, we did not find any association between age and the changes in either the articular processes joints or the intertransverse joints.^22^, ^44^, ^45^ We described the articular process joints and transverse intervertebral joints using six grades. Three of these grades related to the level of fusion of the joint and the other grades were related to new bone formation/sclerosis. We aimed to include the spectrum of the pathologic and congenital findings and to separate congenital fusion (no sclerosis and/or new bone formation) from osteoarthritis (sclerosis and/or new bone formation present). This scale is different to that used in other studies.^6^, ^44^, ^45^ Including a grading scale for the severity of modelling in our grading scheme may have been beneficial and allowed for better identification of osteoarthritis; however, due to the variation in image grade between horses, image quality and resolution were not consistent in all cases to allow such grading.

Most studies describe joint enlargement, as well as periarticular new bone when diagnosing osteoarthritis of the articular process joints.^6^ We found joints smaller than other lumbar articular process joints in the same horse, often with partial or complete fusion. These small joints were frequently seen in combination with a fusion of the intertransverse joints. We speculate that these small, fused joints, represented a congenital finding rather than a degenerative change. The difference between a smooth fused joint, with minimal osseous modelling and an irregular fused or unfused joint, is important in differentiating congenital from degenerative lesions. There is a lack of understanding of the relationships between anatomical variation, congenital abnormality, degenerative/pathological changes and the clinical manifestation of these.

We found intertransverse joints present from the mid‐lumbar spine caudal, with all horses having intertransverse joints at L5‐6 and L6‐S1. The presence of intertransverse joints in the lumbar spine at L5‐6 and L6‐S1 has been described in foals.^46^ Consistent with our findings in horses and ponies over 6 months, intertransverse joints were also found in a smaller number of foals at L4‐5.^46^ One out of 10 foals, was found to have an incomplete intertransverse joint.^46^ Ankylosis or fusion of intertransverse joints has been described previously in adult horses.^41^, ^45^ In this study, we found a correlation between the fusion of the articular process joints and the fusion of the caudal lumbar intertransverse joints at both L5‐6 and L6‐S1 and similar to previous studies, we found sacralisation of the last lumbar vertebra in a small number of cases.^44^, ^47^

The shape of the last lumbar vertebra has previously been linked to certain breeds, with Warmblood horses reportedly having a higher prevalence of ‘sacrum‐shaped’ last lumbar vertebra and Arabs more commonly having a ‘lumbar‐shaped’ last lumbar vertebra.^3^ However, in this study, Warmblood or Warmblood crosses more commonly had a ‘lumbar‐shaped’ last lumbar vertebra. The breed association with the shape of the last lumbar vertebra and the significance this may have on clinical conditions in this region warrants further research.

A high prevalence (35%) of spina bifida has been reported in horses with pain localised to the lumbosacroiliac region^32^ compared with the low prevalence (2.7%) found in a cadaver population of horses where pain was not localised to this region.^4^ In our study, we found spina bifida in the lumbar and sacral spine in nine horses; however, all of the lumbar spine was not assessed in most cases in this case series and a higher prevalence may have been present. In this population, an association between spina bifida and sacroiliac joint osteoarthritis, intervertebral disc disease or ventral spondylosis was not found. We speculate that the presence of spina bifida occulta in the lumbar or sacral spine in most horses is not clinically significant; however, recognise the limitation of the small number of cases with spine bifida within this case series.

Low case numbers and the lack of standardisation of the imaged regions reduce the strength of the conclusions that can be drawn from this study. Not all horses that underwent CT scanning had complete diagnostic evaluations, including diagnostic anaesthesia and imaging. This reflects the nature of clinical practice and highlights the need for a prospective standardised study. This would enhance our ability to discern the clinical significance of the CT imaging findings. Image quality for clinical cases was generally lower than those obtained *post‐mortem* and following abdominal and pelvic evisceration and dissection and represents a limitation of CT for the caudal spine and pelvis in a clinical setting. Low image grade limited the ability to assess relevant structures in all cases. We anticipate that image quality will improve with technological advances and as more powerful CT scanners become available.

Several drawbacks to performing caudal spine and pelvic CT are evident. The requirement for GA remains a barrier for owners and owners should be advised of the risks of GA. This study provides evidence that the risk of morbidity or mortality is low for this procedure as all horses survived without incident, but the number of subjects was small. The procedure is also costly, and for some owners, this cost is prohibitive. We recommend the procedure for horses that have failed to respond to treatment of a condition diagnosed in the caudal spine or pelvis previously and for horses where a diagnosis cannot be made through traditional investigations.

The population for this study was a convenience sample and a power calculation to determine the population size was not performed. Calculating a sample size is important for ensuring statistical power and the ability to detect meaningful effects. When using nonprobability sampling, calculating a sample size may be irrelevant, as it is likely to produce results that cannot be generalised to the larger population, thus hindering statistical conclusions. It is important to recognise that the population in this study does not represent the general population, which is likely to have resulted in selection bias and limits the ability to make broader statistical conclusions.

This study demonstrates that CT is a useful and powerful diagnostic imaging tool for the caudal spine and pelvis. The main advantage of CT of the equine pelvis is the ability to produce cross‐sectional images without superimposition of other anatomical structures. Through increased use, development of more powerful CT machines, and improved evidence base with prospective standardised trials, CT will continue to grow as an important imaging modality for this region. When installing wide‐bore CT machines, careful consideration of the design and construction of the infrastructure should enable caudal spine and pelvic CT in full‐size adult horses.

## FUNDING INFORMATION

None.

## CONFLICT OF INTEREST STATEMENT

The authors declare no conflict of interest.

## AUTHOR CONTRIBUTIONS

**Nadine Kristina Elise Ogden:** Writing – original draft; methodology; investigation; conceptualization. **Katja Winderickx:** Writing – review and editing; investigation. **Alison Bennell:** Writing – original draft. **John David Stack:** Writing – review and editing; investigation; supervision; conceptualization.

## DATA INTEGRITY STATEMENT

Nadine Ogden had full access to all the data in the study and takes responsibility for the integrity of the data and the accuracy of data analysis.

## ETHICAL ANIMAL RESEARCH

Research ethics committee oversight not required for this journal: a retrospective analysis of clinical data.

## INFORMED CONSENT

Horse owners gave consent for the use of their horse data in research in general but not this study explicitly.

## Supporting information


**Figure S1.** CT images (in bone window) demonstrating an example of a ‘lumbar‐shaped’ (A) and ‘sacral‐shaped’ (B) last lumbar vertebra.

**Figure S2.** Flowchart outlining case inclusion. Abbreviations; computer tomography (CT).

**Figure S3.** Cluster column and line combination chart demonstrating the FOV of the CT scans.
Abbreviations; tuber coxa/e (TuC), greater trochanter (GrT), coxofemoral joint/s (CFJ).

**Figure S4.** CT images (in bone window) demonstrating ring artefact (A) and Poisson noise (B).

**Figure S5** Cluster column showing the anatomical variations of the intertransverse joints (dark gray bars) and the articular process joint (grey bars) of the lumbar spine.

**Figure S6.** Cluster column chart demonstrating the variations of inclination of the transverse processes.

**Figure S7.** Cluster column chart demonstrating the variations of impingement of the transverse processes.

**Figure S8.** Cluster column chart demonstrating the variations of inclination of the dorsal spinous process.

**Figure S9.** Cluster column chart demonstrating the variations of dorsal spinous process impingement.

**Figure S10.** Cluster column chart summarising the variations of sacral fusion.

**Table S1.** Sacral fusion table.

**Video S1.** Video of CT acquisition in a 560 kg horse at Hospital 1.

## Data Availability

The data that support the findings of this study are available from the corresponding author upon reasonable request: Open sharing exemption granted by the editor due to lack of provision in the owner consent process.
