# Supplementary material for: Computed tomography of the equine caudal spine and pelvis: Technique, image quality and anatomical variation in 56 clinical cases (2018–2023)
Source: Equine Vet J. 2024 Oct 10;57(5):1265–78. doi: 10.1111/evj.14422 (PMC12326906; doi:10.1111/evj.14422)

Equine Veterinary Journal

Figure S1: CT images (in bone window) demonstrating an example of a ‘lumbar-shaped’ (A) and ‘sacral-shaped’ (B) last lumbar vertebra.

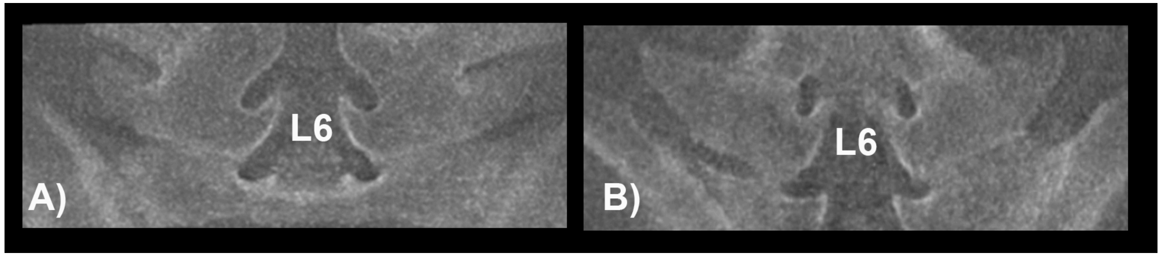

Supplement: Supplementary file 1 — Figure S1. CT images (in bone window) demonstrating an example of a ‘lumbar‐shaped’ (A) and ‘sacral‐shaped’ (B) last lumbar vertebra. [file EVJ-57-1265-s004.pdf]
