# Supplementary figures and images for: Computed tomography of the equine caudal spine and pelvis: Technique, image quality and anatomical variation in 56 clinical cases (2018–2023)
Source: Equine Vet J. 2024 Oct 10;57(5):1265–78. doi: 10.1111/evj.14422 (PMC12326906; doi:10.1111/evj.14422)

**Figure S2:** Flowchart outlining case inclusion. Abbreviations; computer tomography (CT).

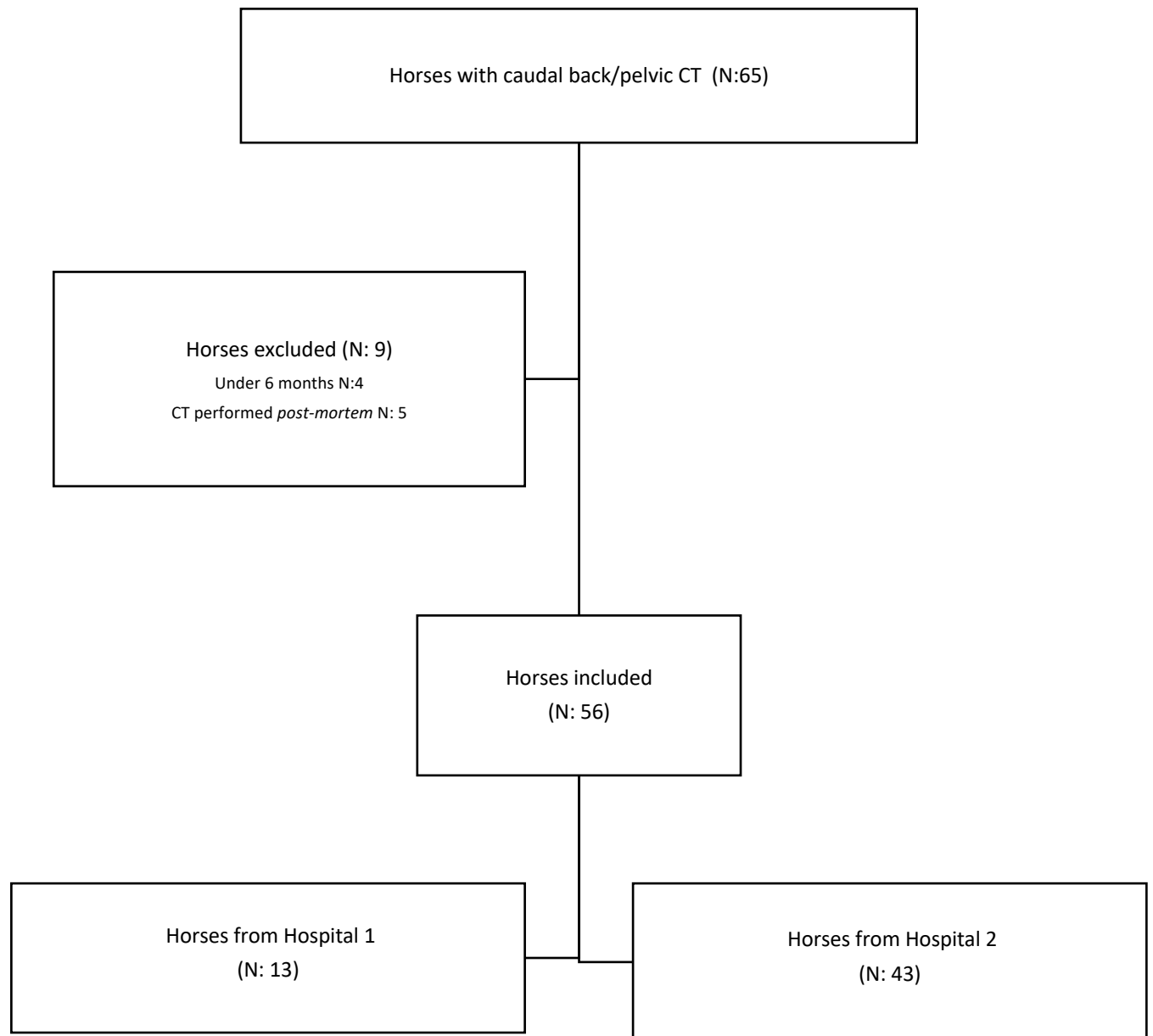

Supplement: Supplementary file 2 — Figure S2. Flowchart outlining case inclusion. Abbreviations; computer tomography (CT). [file EVJ-57-1265-s006.pdf]

**Figure S3:** Cluster column and line combination chart demonstrating the FOV of the CT scans.

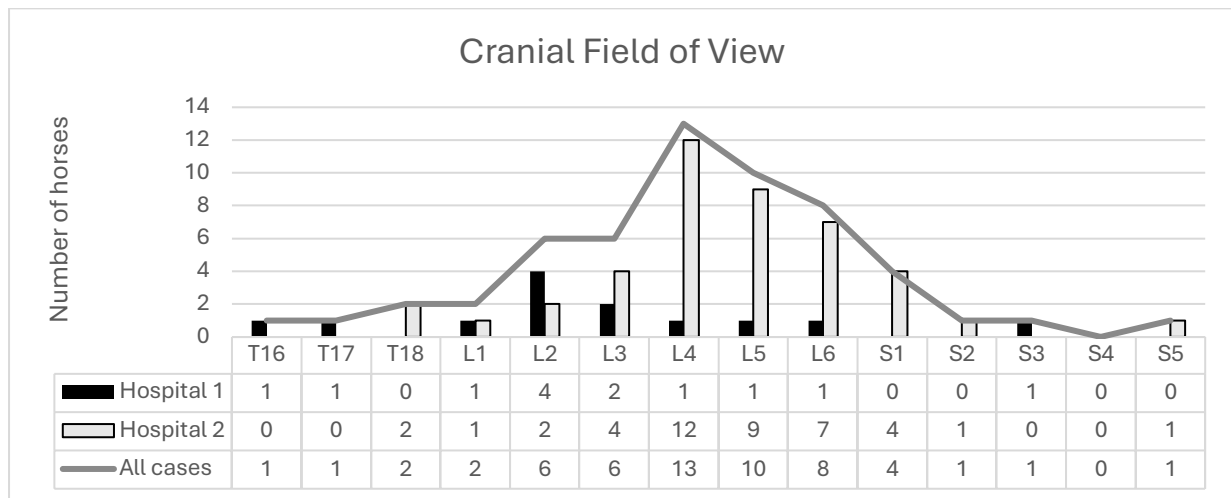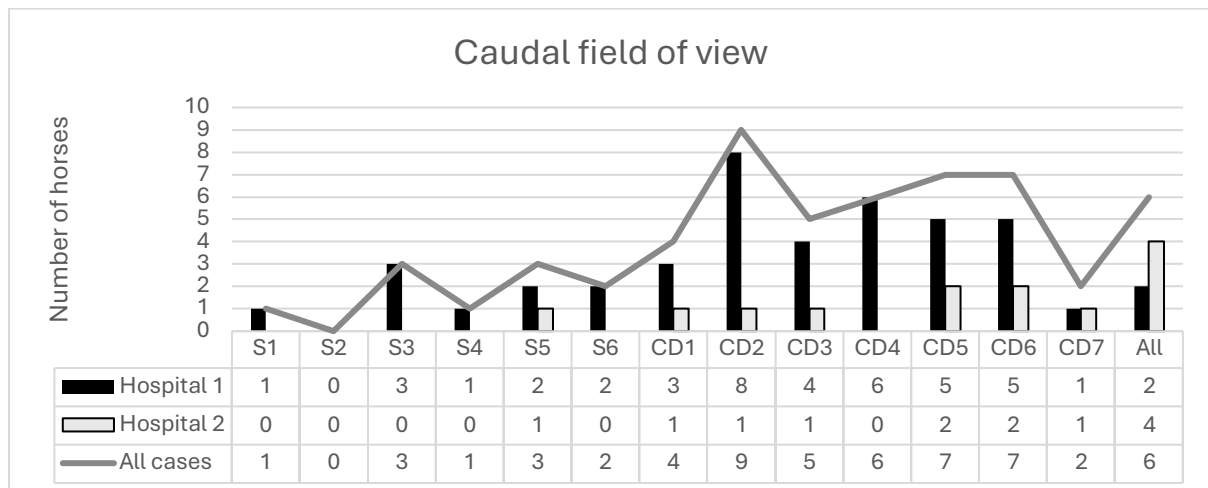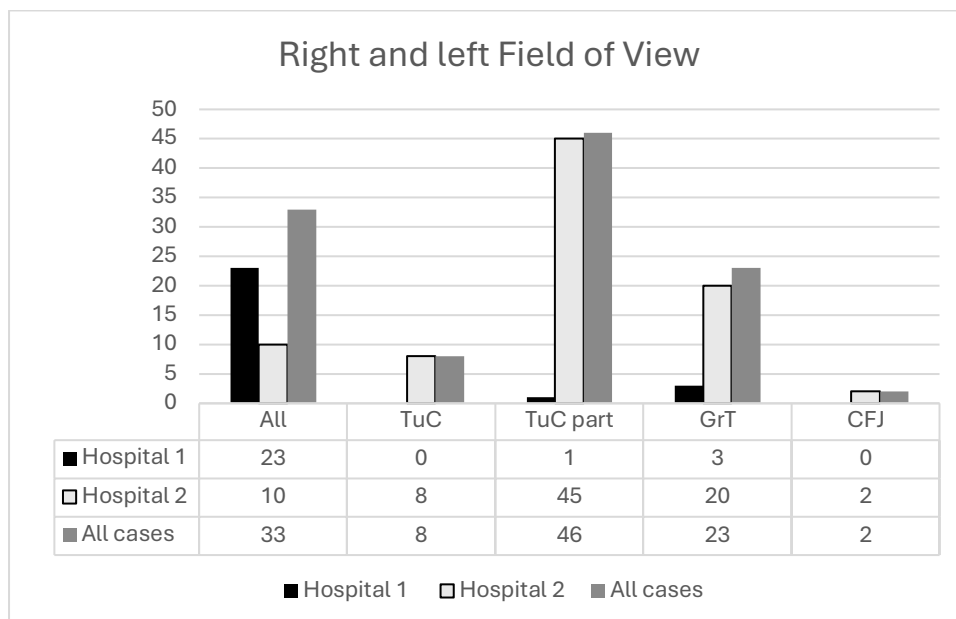

Supplement: Supplementary file 3 — Figure S3. Cluster column and line combination chart demonstrating the FOV of the CT scans. Abbreviations; tuber coxa/e (TuC), greater trochanter (GrT), coxofemoral joint/s (CFJ). [file EVJ-57-1265-s008.pdf]

**Figure S4:** CT images (in bone window) demonstrating ring artefact (A) and Poisson noise (B).

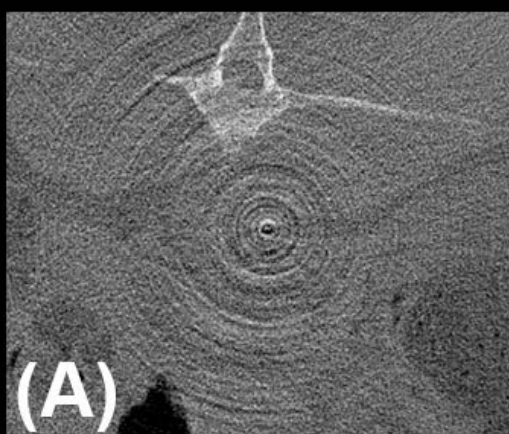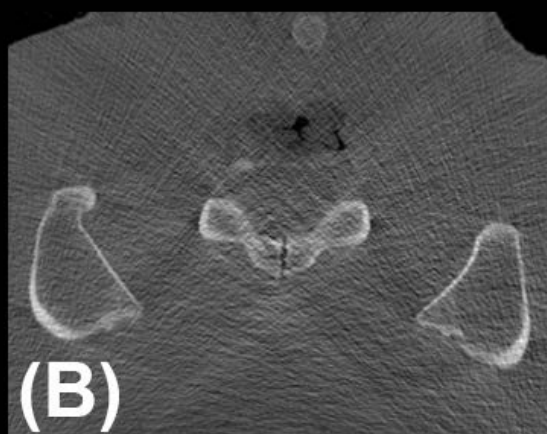

Supplement: Supplementary file 4 — Figure S4. CT images (in bone window) demonstrating ring artefact (A) and Poisson noise (B). [file EVJ-57-1265-s012.pdf]

**Figure S7:** Transverse process impingement and fusion.

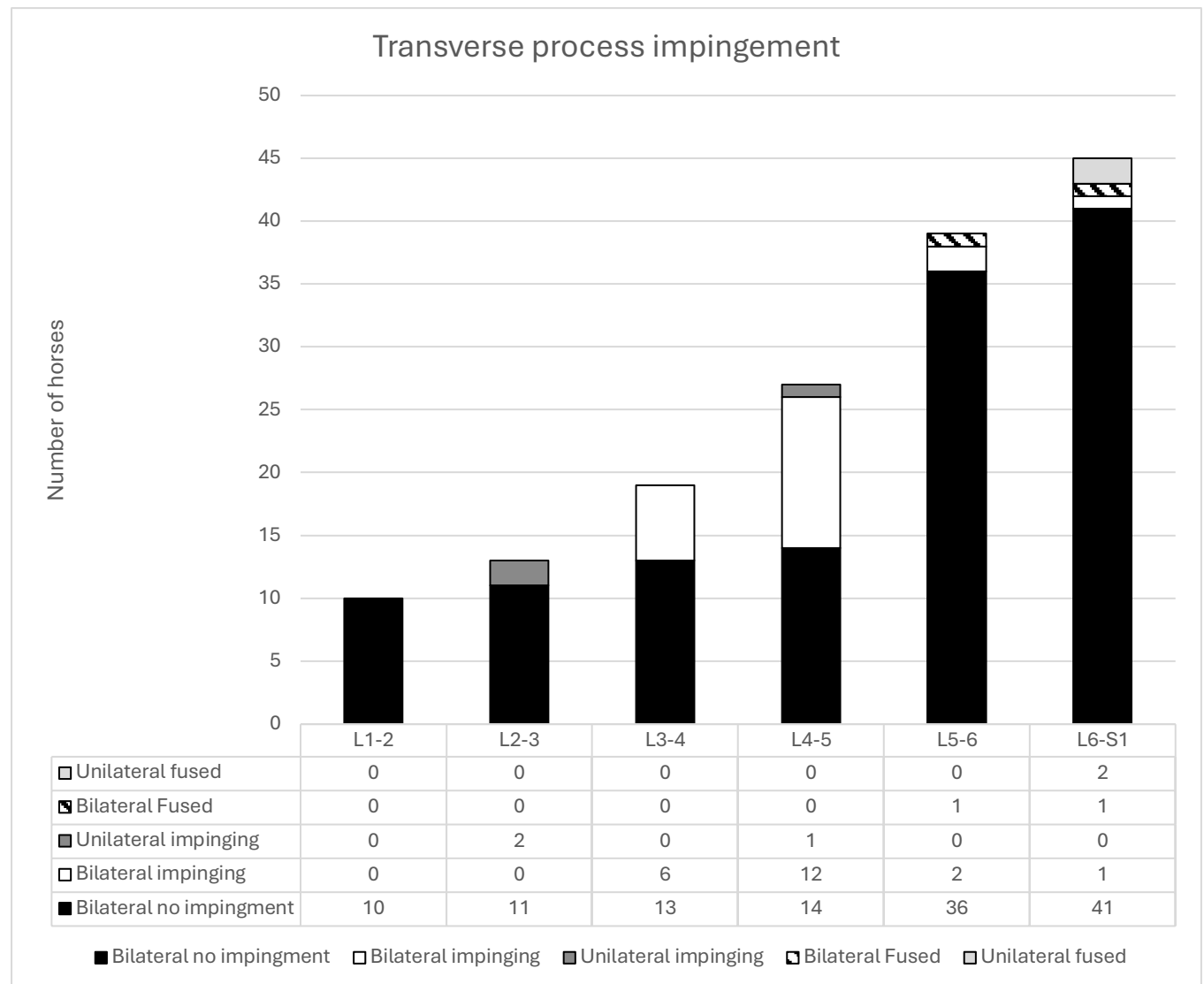

Supplement: Supplementary file 7 — Figure S7. Cluster column chart demonstrating the variations of impingement of the transverse processes. [file EVJ-57-1265-s005.pdf]

**Figure S8:** Dorsal spinous process inclination.

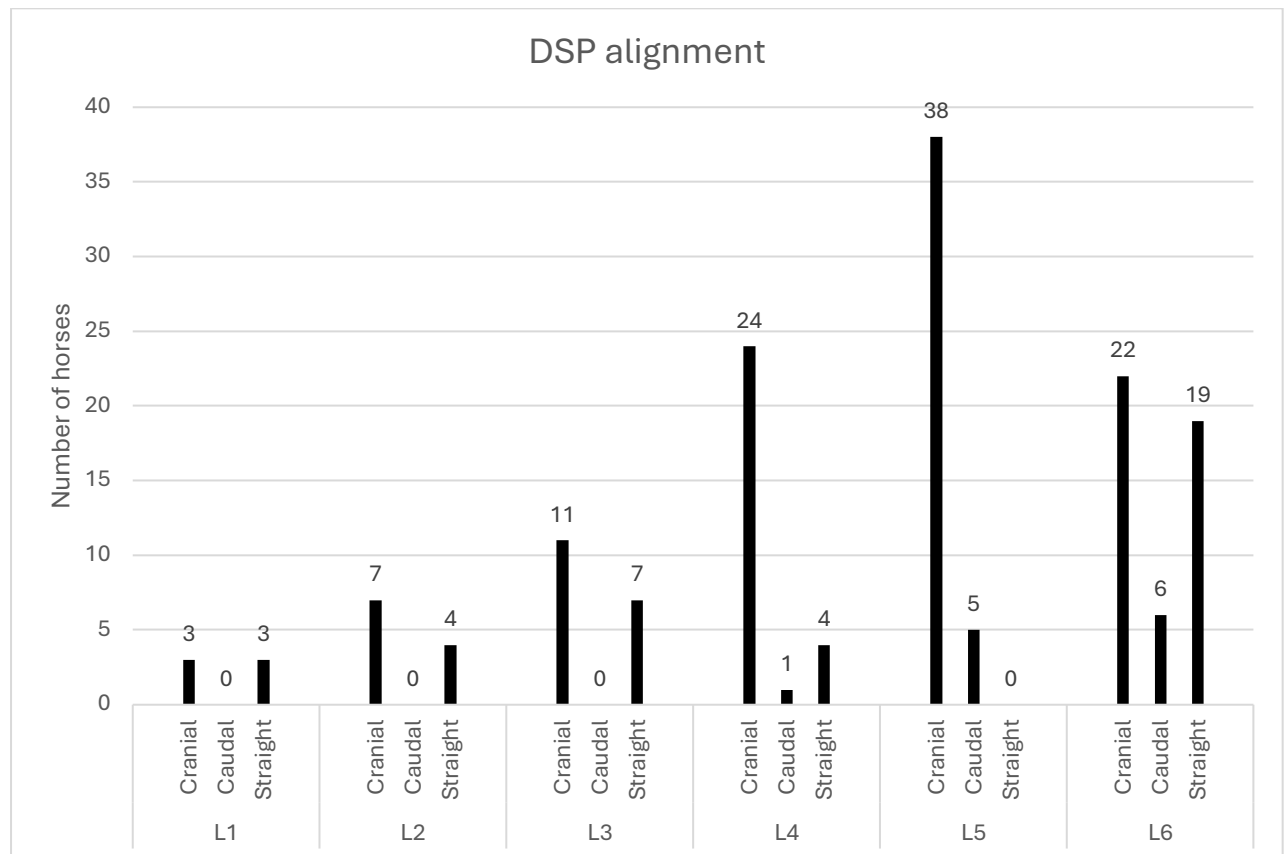

Supplement: Supplementary file 8 — Figure S8. Cluster column chart demonstrating the variations of inclination of the dorsal spinous process. [file EVJ-57-1265-s007.pdf]

**Figure S9:** Dorsal spinous process impingement.

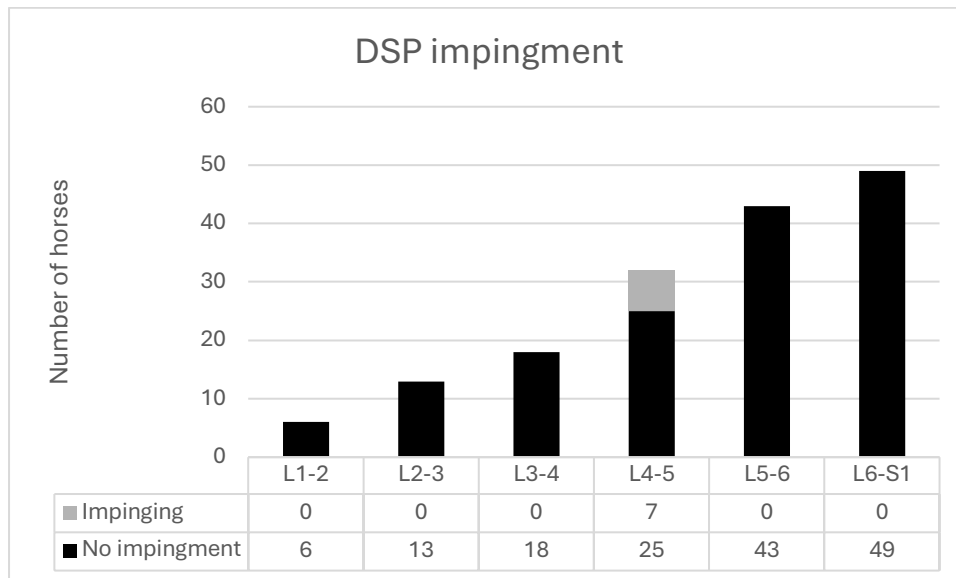

Supplement: Supplementary file 9 — Figure S9. Cluster column chart demonstrating the variations of dorsal spinous process impingement. [file EVJ-57-1265-s003.pdf]

**Figure S10:** Sacral dorsal spinous process fusion.

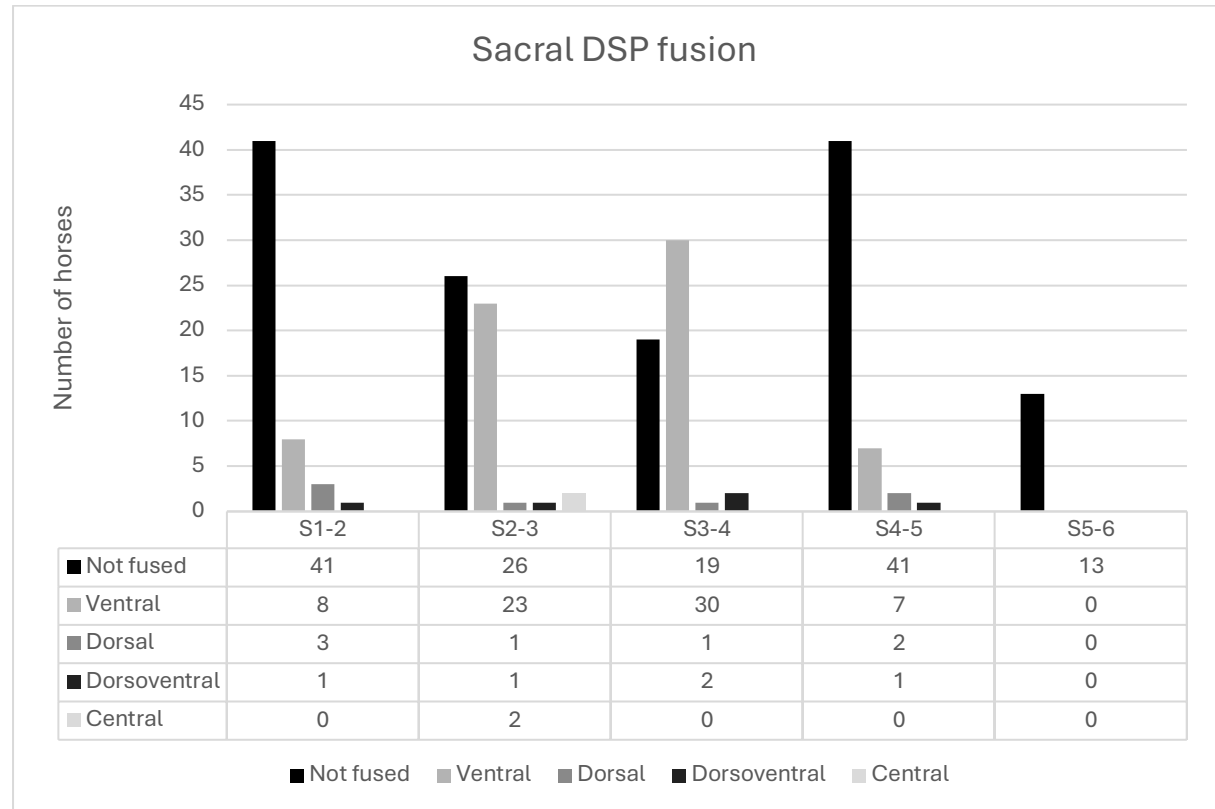

Supplement: Supplementary file 10 — Figure S10. Cluster column chart summarising the variations of sacral fusion. [file EVJ-57-1265-s002.pdf]
