# Supplementary material for: Computed tomography of the equine caudal spine and pelvis: Technique, image quality and anatomical variation in 56 clinical cases (2018–2023)
Source: Equine Vet J. 2024 Oct 10;57(5):1265–78. doi: 10.1111/evj.14422 (PMC12326906; doi:10.1111/evj.14422)

Equine Veterinary Journal BEVA

Figure S5: Cluster column showing the anatomical variations of the intertransverse joints (black bars) and the articular process joint (grey bars) of the lumbar spine.

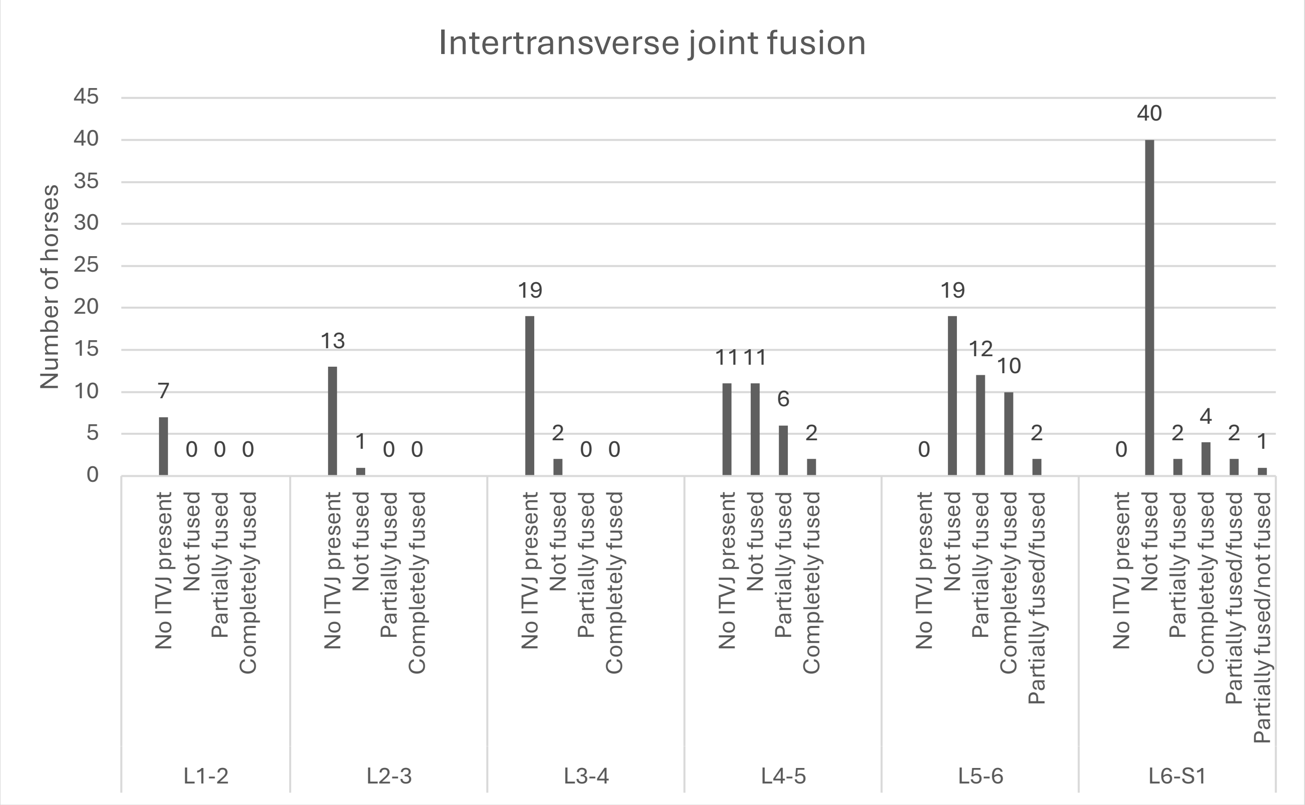

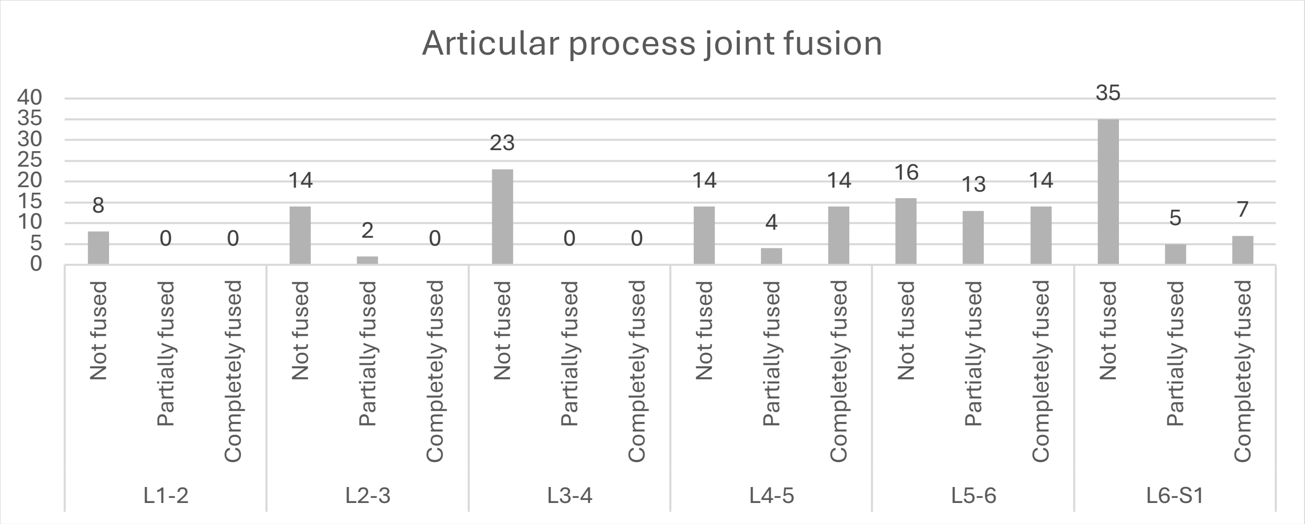

Supplement: Supplementary file 5 — Figure S5 Cluster column showing the anatomical variations of the intertransverse joints (dark gray bars) and the articular process joint (grey bars) of the lumbar spine. [file EVJ-57-1265-s010.pdf]
