# Supplementary material for: Computed tomography of the equine caudal spine and pelvis: Technique, image quality and anatomical variation in 56 clinical cases (2018–2023)
Source: Equine Vet J. 2024 Oct 10;57(5):1265–78. doi: 10.1111/evj.14422 (PMC12326906; doi:10.1111/evj.14422)

Equine Veterinary Journal BEVA

Figure S6: Transverse spinous process inclination.

*One horse had a caudal alignment of the transverse process on the right side and a straight alignment on the left side, this horse has been counted twice in both the caudal and straight group.

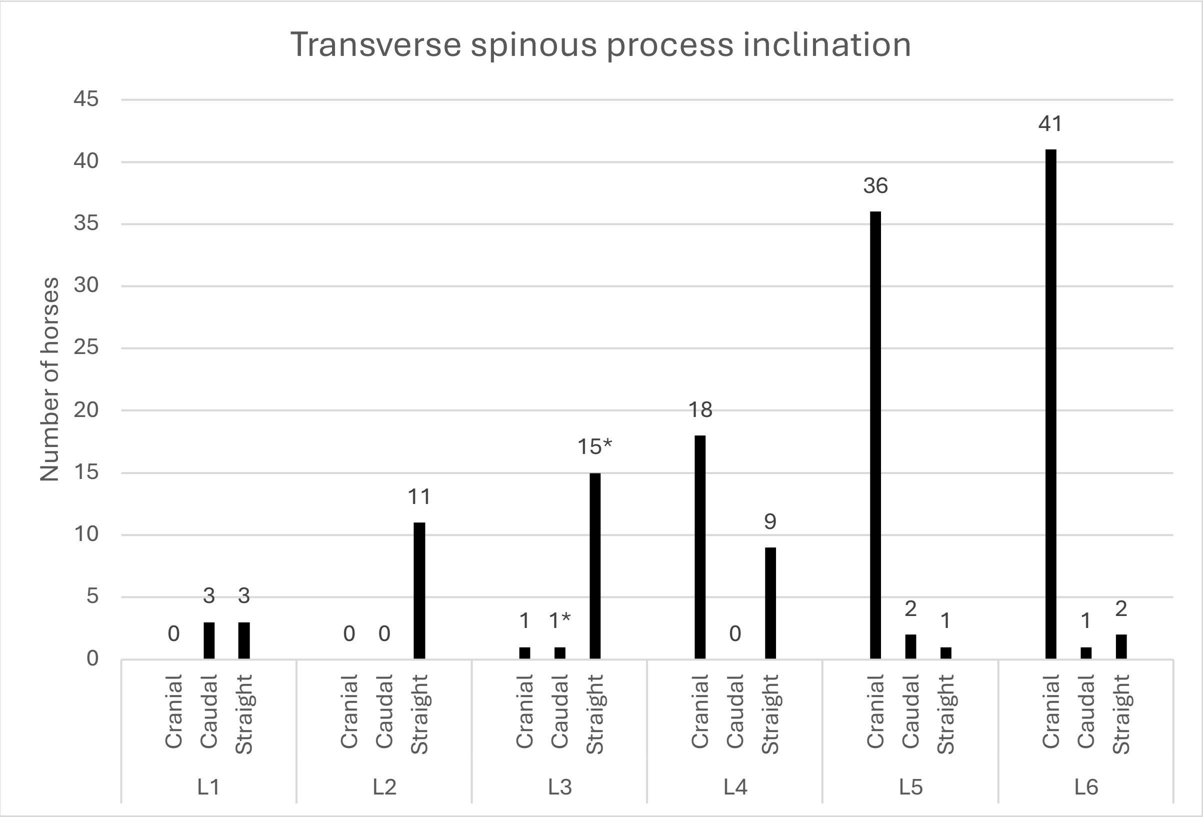

Supplement: Supplementary file 6 — Figure S6. Cluster column chart demonstrating the variations of inclination of the transverse processes. [file EVJ-57-1265-s011.pdf]
