# Supplementary material for: Computed tomography of the equine caudal spine and pelvis: Technique, image quality and anatomical variation in 56 clinical cases (2018–2023)
Source: Equine Vet J. 2024 Oct 10;57(5):1265–78. doi: 10.1111/evj.14422 (PMC12326906; doi:10.1111/evj.14422)
Supplement: Supplementary file 11 — Table S1. Sacral fusion table. [file EVJ-57-1265-s001.pdf]

Equine Veterinary Journal BEVA

Table S2: Sacral dorsal spinous process fusion (%).

Variable	Mean	StDev	Minimum	Median	Maximum
S1-2 Ventral	31.00	16.63	12.00	26.50	59.00
S2-3 Ventral	37.23	22.67	13.00	28.50	98.00
S3-4 Ventral	36.76	18.16	11.00	32.00	84.00
S4-5 Ventral	28.86	4.78	19.00	29.00	33.00
S1-2 Dorsal	23.67	4.73	20.00	22.00	29.00
S2-3 Dorsal	37.000	*	37.000	37.000	37.000
S3-4 Dorsal	39.000	*	39.000	39.000	39.000
S4-5 Dorsal	33.00	9.90	26.00	33.00	40.00
S1-2 Dorsoventral (dorsal)	10.000	*	10.000	10.000	10.000
S1-2 Dorsoventral (ventral)	10.000	*	10.000	10.000	10.000
S2-3 Dorsoventral (dorsal)	14.000	*	14.000	14.000	14.000
S2-3 Dorsoventral (ventral)	33.000	*	33.000	33.000	33.000
S3-4 Dorsoventral (dorsal)	30.50	13.44	21.00	30.50	40.00
S3-4 Dorsoventral (ventral)	18.0	17.0	6.0	18.0	30.0
S4-5 Dorsoventral (dorsal)	20.000	*	20.000	20.000	20.000
S4-5 Dorsoventral (ventral)	24.000	*	24.000	24.000	24.000
S2-3 Central	76.0	19.8	62.0	76.0	90.0
